# Comparative Genomics and Proteomic Analysis of Assimilatory Sulfate Reduction Pathways in Anaerobic Methanotrophic Archaea

**DOI:** 10.3389/fmicb.2018.02917

**Published:** 2018-12-03

**Authors:** Hang Yu, Dwi Susanti, Shawn E. McGlynn, Connor T. Skennerton, Karuna Chourey, Ramsunder Iyer, Silvan Scheller, Patricia L. Tavormina, Robert L. Hettich, Biswarup Mukhopadhyay, Victoria J. Orphan

**Affiliations:** ^1^Division of Geological and Planetary Sciences, California Institute of Technology, Pasadena, CA, United States; ^2^Ronald and Maxine Linde Center for Global Environmental Science, California Institute of Technology, Pasadena, CA, United States; ^3^Department of Biochemistry, Virginia Tech, Blacksburg, VA, United States; ^4^Chemical Sciences Division, Oak Ridge National Laboratory, Oak Ridge, TN, United States; ^5^Graduate School of Genome Science and Technology, University of Tennessee, Knoxville, Knoxville, TN, United States; ^6^Biocomplexity Institute, Virginia Tech, Blacksburg, VA, United States; ^7^Virginia Tech Carilion School of Medicine, Virginia Tech, Blacksburg, VA, United States

**Keywords:** sulfur pathway, sulfate reduction, anaerobic oxidation of methane, ANME, syntrophy, sulfate adenylyltransferase, APS/PAPS Reductase, sulfite reductase

## Abstract

Sulfate is the predominant electron acceptor for anaerobic oxidation of methane (AOM) in marine sediments. This process is carried out by a syntrophic consortium of anaerobic methanotrophic archaea (ANME) and sulfate reducing bacteria (SRB) through an energy conservation mechanism that is still poorly understood. It was previously hypothesized that ANME alone could couple methane oxidation to dissimilatory sulfate reduction, but a genetic and biochemical basis for this proposal has not been identified. Using comparative genomic and phylogenetic analyses, we found the genetic capacity in ANME and related methanogenic archaea for sulfate reduction, including sulfate adenylyltransferase, APS kinase, APS/PAPS reductase and two different sulfite reductases. Based on characterized homologs and the lack of associated energy conserving complexes, the sulfate reduction pathways in ANME are likely used for assimilation but not dissimilation of sulfate. Environmental metaproteomic analysis confirmed the expression of 6 proteins in the sulfate assimilation pathway of ANME. The highest expressed proteins related to sulfate assimilation were two sulfite reductases, namely assimilatory-type low-molecular-weight sulfite reductase (alSir) and a divergent group of coenzyme F_420_-dependent sulfite reductase (Group II Fsr). In methane seep sediment microcosm experiments, however, sulfite and zero-valent sulfur amendments were inhibitory to ANME-2a/2c while growth in their syntrophic SRB partner was not observed. Combined with our genomic and metaproteomic results, the passage of sulfur species by ANME as metabolic intermediates for their SRB partners is unlikely. Instead, our findings point to a possible niche for ANME to assimilate inorganic sulfur compounds more oxidized than sulfide in anoxic marine environments.

## Introduction

The anaerobic oxidation of methane (AOM) is an important biogeochemical process in the global carbon cycle, and is the primary sink for methane in anoxic ocean sediments (Reeburgh, 2007). The diffusion of seawater sulfate into sediments serves as the major electron acceptor for this process, fueling a syntrophic association between uncultured anaerobic methanotrophic archaea (ANME) and sulfate-reducing bacteria (SRB) in regions where methane seepage occurs. Since the discovery of the AOM syntrophy (Hinrichs et al., 1999; Boetius et al., 2000; Orphan et al., 2001), a number of hypotheses have been proposed on how ANME and SRB function together (Knittel and Boetius, 2009), but they have not been fully resolved.

Diffusible intermediates such as hydrogen, formate, or acetate could be exchanged between ANME and SRB to allow energy metabolism of AOM coupled to sulfate reduction (Valentine and Reeburgh, 2000; Moran et al., 2008; Alperin and Hoehler, 2009). However, these hypotheses are inconsistent with results from incubation experiments (Nauhaus et al., 2002, 2005; Meulepas et al., 2009; Wegener et al., 2016). More recent work has suggested that ANME could be syntrophically coupled to SRB via direct interspecies electron transfer (Meyerdierks et al., 2010; McGlynn et al., 2015; Wegener et al., 2015; Scheller et al., 2016; Skennerton et al., 2017). Alternatively, ANME (in particular ANME-2a and ANME-2c lineages) have been hypothesized to couple methane oxidation to sulfate reduction, releasing zero-valent sulfur which is subsequently disproportionated by SRB (Milucka et al., 2012). Recent attempts to culture the syntrophic SRB partners of ANME using zero-valent sulfur were unsuccessful (Wegener et al., 2016). Furthermore, a genetic and biochemical basis for dissimilatory sulfate reduction by ANME is currently lacking. Aside from members of the distantly related *Archaeoglobales* (Pereira et al., 2011), no other euryarchaeotal group has been shown to have the genetic capability for energy conservation through dissimilatory sulfate reduction.

Components of the assimilatory sulfate reduction pathway were found previously in ANME-1 and ANME-2c lineages, suggesting the genomic potential for biochemical transformation of oxidized forms of sulfur (Meyerdierks et al., 2010; Krukenberg et al., 2018). On the other hand, marker genes or proteins for canonical dissimilatory sulfate reduction have not been detected in ANME (Meyerdierks et al., 2010; Milucka et al., 2013; Wang et al., 2014; Krukenberg et al., 2018). All cultured methanogens to date can use sulfide for biosynthesis (Liu et al., 2012). Given that ANME live in highly sulfidic environments, it stands to reason that they too would preferentially assimilate sulfide rather than invest energy in sulfate assimilation. However, the genomic capacity for sulfur metabolism has not been fully explored in different ANME lineages.

An important step in sulfate reduction is the six electron reduction of sulfite to sulfide by assimilatory or dissimilatory sulfite reductases. Sulfite reductases can be classified into different phylogenetic groups and are found in the genomes of methanogens (Dhillon et al., 2005; Loy et al., 2008; Susanti and Mukhopadhyay, 2012). The assimilatory-type low-molecular-weight sulfite reductase (alSir, also called Group I Dsr-LP) have been biochemically characterized and shown to reduce sulfite (Moura et al., 1982). While alSir is not involved in dissimilatory sulfur metabolism in the bacteria *Desulfuromonas acetoxidans*, its physiological role remains unclear (Moura et al., 1986; Moura and Lino, 1994). Another sulfite reductase, coenzyme F_420_-dependent sulfite reductase (Fsr), was more recently characterized in *Methanocaldococcus jannaschii* (Johnson and Mukhopadhyay, 2005). Fsr is a fusion protein consisting of the beta subunit of the F_420_H_2_ dehydrogenase at the N-terminus and a sulfite reductase at the C-terminus, together couple F_420_H_2_ oxidation to sulfite reduction (Johnson and Mukhopadhyay, 2005). When Fsr from *M. jannaschii* was heterologously expressed in sulfite-sensitive *Methanococcus maripaludis*, *M. maripaludis* was able to tolerate and assimilate sulfite as the sole sulfur source (Johnson and Mukhopadhyay, 2008). Both alSir and Fsr were found in ANME-1 and ANME-2c genomes and expressed in the metatranscriptome (Hallam et al., 2004; Susanti and Mukhopadhyay, 2012; Krukenberg et al., 2018), but their physiological roles remain unknown.

Here we focus on identifying potential sulfur pathway genes in ANME, building from a collection of newly sequenced genomes to cover different lineages. Our genome observations were then combined with metaproteomics and microcosm experiments to gain further insight into the role of sulfur on ANME and their partner SRB. The capacity for sulfur usage by different ANME lineages is an important aspect to understanding energy conservation and syntrophy in AOM.

## Materials and Methods

### Genome Retrieval of Different ANME Lineages

Genomic database of ANME consisted of 3 newly sequenced genomes, as well as previously published data to cover ANME lineages ANME-1b, ANME-2a, ANME-2b, ANME-2c, and *Candidatus* Methanoperedens (formerly known as ANME-2d).

A new ANME-1b genome (CONS3730B06UFb1), estimated to be 90% complete and 2.4% contamination by CheckM software package v1.0.6 using the taxonomy workflow and the Euryarchaeota set of markers (Parks et al., 2015), was obtained from methane seep sediment at Hydrate Ridge, United States (ID 3730; Supplementary Table 3) using activity-based cell sorting method in a previous study (Hatzenpichler et al., 2016). In addition to 16S rRNA gene analysis of multiple displacement amplified products, a 300 bp insert standard shotgun library was constructed and sequenced using the Illumina NextSeq platform, All general aspects of library construction and sequencing performed at the JGI can be found at http://www.jgi.doe.gov. BBTools software tools^1^ was used to remove Illumina artifacts, PhiX, reads with more than one “N” or with quality scores (before trimming) averaging less than 8 or reads shorter than 51 bp (after trimming), reads with > 95% identity mapped to masked versions of human, cat, and dog references. Then, reads with high k–mer coverage (>100× average k–mer depth) were normalized and error corrected to an average depth of 100×. Reads with an average k–mer depth of less than 2× were removed. These reads were assembled using SPAdes (version 3.6.2) (Bankevich et al., 2012), and any contigs with length is <1 kbp were discarded. A final binning was performed based on GC content (Laczny et al., 2015), which only showed 1 genome bin containing all contigs in this sample. This new ANME-1b genome was used in our analysis in addition to previously published fosmid sequences of this lineage (Meyerdierks et al., 2010) and reconstructed genomes under NCBI GenBank assembly accessions GCA_003194425.1 and GCA_003194435.1 (Krukenberg et al., 2018).

For ANME-2a lineage, we used the previously published genome under IMG Submission ID 36455 (Wang et al., 2014). A new ANME-2b genome (HR1), estimated to be 95.73% complete with 0.06% contamination by CheckM software package v1.0.6 (Parks et al., 2015), of this previously unsequenced lineage was obtained from a methane seep bulk metagenome from sediment ID 5133, recovered from Hydrate Ridge, United States (Supplementary Table 3) (Marlow et al., 2016; Trembath-Reichert et al., 2016). DNA was extracted using the UltraClean Soil DNA isolation kit (Mo Bio Laboratories, Carlsbad, CA, United States) from ∼0.5 g of bulk methane seep sediment, sequenced using the Illumina HiSeq platform and processed as described previously (Marlow et al., 2016).

A new ANME-2c genome (S7142MS2), estimated to be 89.15% complete with 6.04% contamination by CheckM software package v1.0.6 (Parks et al., 2015), was obtained from sediment ID 7142 collected from the Santa Monica Basin (Supplementary Table 3) by bulk metagenome sequencing. DNA from methane seep sediment incubation #7142 (∼2 ml) was extracted using the MoBio Powersoil DNA kit (MoBio Laboratories Inc., Carlsbad, CA, United States) according to the manufacturer’s protocol. The paired-end 2 × 150 bp library was prepared using the Nextera XT DNA library preparation kit (Illumina, San Diego, CA, United States), and sequenced on a NextSeq500 (Illumina, San Diego, CA, United States) platform. Bulk metagenome reads were trimmed and quality filtered using Trimmomatic (Bolger et al., 2014) and BBMerge^2^ using default settings. Low-abundance k-mer trimming was applied using the khmer script trim-low-abund.py (Crusoe et al., 2015) using with the K = 20 and C = 30 parameter and assembled with Metaspades version 3.9.0 (Nurk et al., 2017) using the default parameters. Scaffolding and gap-filling of the metagenome assembly was performed using the “roundup” mode of FinishM v0.0.7^3^. Population genomes were recovered from the assembled contigs using MetaBat (Kang et al., 2015). ANME sp. S7142MS2 was further refined by removing scaffolds with divergent GC-content, tetranucleotide frequencies or coverage using the outlier method in RefineM v0.0.13^4^. These were used in addition to ANME-2c fosmids under NCBI GenBank ID AY714844 (Hallam et al., 2004) and reconstructed genome under NCBI GenBank assembly accession GCA_003194445.1 (Krukenberg et al., 2018).

For *Ca.* Methanoperedens, published genome data was used from NCBI BioProject PRJNA224116 and PRJNA296416 for *Ca.* Methanoperedens nitroreducens and *Ca.* Methanoperedens sp. BLZ1, respectively (Haroon et al., 2013; Arshad et al., 2015). All other reference sequences used in our analysis were retrieved from databases NCBI Refseq and Integrated Microbial Genomes with Microbiome Samples (IMG/MER) (Markowitz et al., 2012; Pruitt et al., 2012).

### Bioinformatic Analyses of Sulfur Pathways in ANME and Methanogens

Sulfur pathway genes were first identified using BLASTP (*E*-value cut-off of 1e1) to a custom protein database consisting of ANME and methanogen genomes listed in Supplementary Table 1. The protein sequences were then aligned using Clustal Omega (Sievers et al., 2011) and all homologs were identified through an iterative alignment evaluation based on characterized proteins and manual selection. The results were imported into the ARB package (Ludwig et al., 2004) and checked for misalignments. After excluding columns with gaps as the most common occurring character, 416 and 270 aligned positions were used for phylogenetic analysis for *cysN*/EF-1A/EF-Tu and *cysD*, respectively. For APS/PAPS reductases, since some homologs have acquired extra N- or C-terminus domains, only 172 aligned amino acids from the central shared region excluding columns with gaps as the most common occurring character were used for phylogenetic analysis. The extra N-/C-terminus 4Fe-4S domains were identified based on conserved cysteine cluster binding motif (CX_2_CX_2_CX_3_C), and the cysteine desulfurylase domains were identified using InterPro online 69.0 (Finn et al., 2017). For sulfite reductases, since different groups have acquired extra domains for flavin or iron-sulfur cluster binding, or F_420_H_2_ oxidation, only the shared catalytic and siroheme binding region with 224 amino acid residues was used for phylogenetics. The trees were built using MrBayes v.3.2.1 (Ronquist et al., 2012) with a mixed amino acid model burn-in set to 25% and stop value set to 0.01, and edited using iTOL (Letunic and Bork, 2016).

For protein homology modeling of Group II Fsr, ANME Fsr sequences were trimmed to contain only the C-terminal sulfite reductase half of the protein as done previously (Johnson and Mukhopadhyay, 2005). Protein structural prediction was performed using I-TASSER online server V4.1 (Zhang, 2008; Roy et al., 2010, 2012) with default parameters. The predicted structure and its most similar template in the Protein Data Bank, the dissimilatory sulfite reductase alpha subunit from *Archaeoglobus fulgidus* (PDB 3mm5 Chain A), were imported and viewed in PyMOL Molecular Graphics System (Delano, 2002).

### Primer Design and Amplification of *fsr* From ANME in Different Methane Seep Samples

DNA extracts used in PCR amplification were obtained from 4 methane seep sediments with the following sediment IDs: 3730, 5059, 5207, 5547 (Supplementary Table 3). Anoxic 0.22 μm filtered bottom seawater was collected on a 2011 R/V Atlantis cruise AT 18–10 to Hydrate Ridge. This seawater was mixed in a 2:1 ratio with the sediment supplied with 0.3 MPa methane headspace and maintained at 10°C in the dark. DNA from the sediment slurries (0.2 g of wet weight sediment) was extracted using the PowerSoil DNA extraction kit (Mo Bio Laboratories Inc., Carlsbad, CA, United States) following the manufacturer’s instructions, with the bead beating option using FastPrep FP120 (Thermo Electron Corporation, Milford, MA, United States) at setting 5.5 for 45 s instead of the 10 min vortex step. Also, DNA was extracted from *Methanococcoides burtonii* cultures using Qiagen DNeasy Blood & Tissue Kit (Qiagen, Hilden, Germany) following manufacturer’s protocol for Gram-positive bacteria.

Degenerate primer sets were designed to study ANME *alSir* and Group II *fsr* in environmental samples (Supplementary Table 4). PCR was performed using the TaKaRa Ex Taq^®^ DNA Polymerase kit (Takara Bio United States, Inc., Mountain View, CA, United States) with the following conditions: 1.0 μl of 10 × buffer, 0.2 μl of dNTP, 0.2 μl of Taq polymerase, 0.2 μl of each forward and reverse primer, 7.2 μl of PCR water, and 1 μl of DNA sample. The cycling conditions were as following: 95°C for 40 s, 40 cycles of 94°C for 20 s, annealing at 59°C for 30 s, extension at 72°C for 100 s, and a final extension step at 72°C for 4 min before cooling down to 4°C. The products were immediately purified using Multiscreen HTS plates (Millipore, Billerica, MA, United States), and cloned using TOPO TA Cloning Kit for Sequencing with pCR4-TOPO Vector and One Shot Top 10 Chemically Competent *Escherichia coli* following manufacturer’s instructions (Life Technologies, Carlsbad, CA, United States). Over 100 transformants were observed on plate with 20 μl of initial cells. Clones were grown overnight in Luria-Bertani medium containing ampicillin as used in the TOPO TA cloning procedure (Life Technologies, Carlsbad, CA, United States). PCR was performed using the NEB Taq Polymerase kit (New England Biolabs, Ipswich, MA, United States) with the following conditions: 2.5 μl of 10 × buffer, 0.55 μl of dNTP, 0.13 μl of Taq polymerase, 0.5 μl of each M13 forward and reverse primer, 20.3 μl of PCR water, and 0.5 μl of cells. The cycling conditions were as following: 95°C for 40 s, 30 cycles of 94°C for 20 s, annealing at 54°C for 45 s, extension at 72°C for 100 s, and a final extension step at 72°C for 4 min before cooling down to 4°C. Sanger sequencing was performed on the resulting PCR products using both M13 forward or reverse primers (Laragen Inc., Culver City, CA, United States).

### Metaproteomic Analysis of ANME Proteins in Methane Seep Sediments

The expression of sulfur pathway genes by ANME was investigated using environmental metaproteomic data from three methane seep samples (sediment IDs 3730, 5133, and 5579; Supplementary Table 3). These samples showed active methane-dependent sulfate reduction, and fluorescence microscopy showed characteristic AOM aggregates. The samples were maintained anaerobically at 4°C under methane headspace in natural seawater in the laboratory prior to subsampling for protein analysis as described previously (Marlow et al., 2016; Skennerton et al., 2017). All chemicals used for sample preparation and mass spectrometry analysis were obtained from Sigma Chemical Co. (St Louis, MO, United States), unless mentioned otherwise. High performance liquid chromatography (HPLC) grade water and other solvents were obtained from Burdick & Jackson (Muskegon, MI, United States).

For protein extraction, 5 g of thawed seep sediments were suspended in 10 ml of detergent lysis buffer and then subjected to cellular lysis as described previously (Chourey et al., 2010). The slurry was cooled down to room temperature and centrifuged for 5 min at 8000 × *g* to settle the sediment. The clear supernatant was transferred to fresh Eppendorf tubes and treated with 100% trichloroacetic acid (TCA) to final concentration of 25% and kept at −20°C overnight. The supernatant was later centrifuged at 21,000 × *g* to obtain a protein pellet, which was subsequently washed with chilled acetone, air dried, and solubilized in a 6 M guanidine buffer as described previously (Chourey et al., 2013; Bagnoud et al., 2016). Protein estimation was carried out using RC/DC protein estimation kit (Bio-Rad Laboratories, Hercules, CA, United States). Protein mix was subjected to trypsin digestion (Promega, Madison, WI, United States), desalted and solvent exchanged as described previously (Thompson et al., 2007). Peptides were stored at −80°C until MS analysis.

Peptide samples (100 μg) were loaded on a biphasic resin packed column [SCX (Luna, Phenomenex, Torrance, CA, United States) and C18 (Aqua, Phenomenex, Torrance, CA, United States)] as described previously (Brown et al., 2006; Thompson et al., 2007), and subjected to a offline wash as described previously (Sharma et al., 2012). Peptide elution, fragmentation and measurements were conducted via an online MudPIT (multi-dimensional protein identification technology) on a nano 2D LC–MS/MS system interfaced with LTQ-Velos Pro MS (Thermo Fisher Scientific, Whaltham, MA, United States) using the parameters as described previously (Sharma et al., 2012; Bagnoud et al., 2016).

**FIGURE 1:**
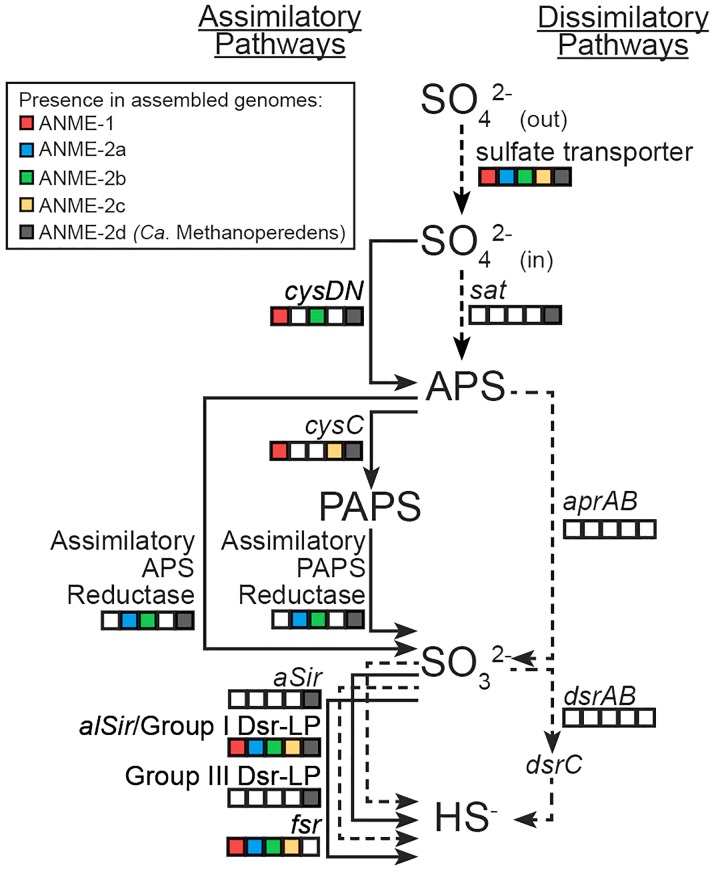
Sulfur assimilatory and dissimilatory pathways in different ANME lineages. Squares are color-filled based on the presence of particular gene(s) in ANME-1/2a/2b/2c and *Ca.* Methanoperedens (ANME-2d). Lines are solid and dotted based on the presence or absence of particular gene(s) in marine ANME lineages that live syntrophically with SRB partners. Putative sulfate transporters or *sat* could be used for either assimilatory or dissimilatory sulfate reduction. Known genes in the dissimilatory pathway (*aprAB, dsrAB* and their membrane complexes) were not identified in any ANME lineage.

We used two approaches to search the proteome database: (1) general bulk expression analysis using a custom methane seep metagenome database as in our previous study (Marlow et al., 2016), and (2) specific search of sulfur pathway genes of ANME using only those protein sequences of interest following an approach outlined previously (Skennerton et al., 2017). The custom sulfur database included those proteins identified in ANME genomes in Figure 1, as well as the Fsr sequences PCR amplified in this study. The MS/MS fragmentation spectra was searched against these two databases using Myrimatch v2.1 algorithm (Tabb et al., 2007). A decoy database of reversed protein sequences and common contaminants from keratin and trypsin was appended to the target database containing sulfur pathway genes from ANME genome bins above. Peptide FDR was set to <1% and a minimum of 1 unique and 1 non-unique peptide was required for protein identification. Normalization of spectral counts was carried out as described previously (Paoletti et al., 2006; Neilson et al., 2013) to obtain normalized spectral counts (nSpC) as described previously (Sharma et al., 2012; Marlow et al., 2016).

### Response of ANME-2a/2c and *Methanococcoides burtonii* to Different Sulfur Compounds

To study the metabolic response of ANME, we tracked methane oxidation rates using ^13^C-labeled CH_4_ to different sulfur amendments. Microcosm experiments were set up using methane seep sediment ID 7142 as described previously (Scheller et al., 2016). Briefly, 5 ml of sediment slurry containing 1 ml of wet sediment in artificial seawater was incubated under a 0.250 MPa CH_4_ headspace containing ca. 4% ^13^CH_4_. Periodically, overlying seawater was sampled anaerobically, centrifuged at 16,000 × *g* for 5 min, and saved at −20°C until analysis using Gasbench II (Thermo Fisher Scientific, Whaltham, MA, United States) coupled to a Delta V Plus IRMS instrument (Thermo Fisher Scientific, Whaltham, MA, United States). ANME-2a and ANME-2c were found to be the most abundant ANME lineages in this sediment sample (Scheller et al., 2016). Polythionate solutions were prepared following a previous described protocol, purified by precipitation with saturated NaCl for 6 times and quantified by dry weight according to the predicted composition (Steudel et al., 1989). Polysulfide solutions were prepared by autoclaving sulfide solutions in an excess of sulfur powder, and the concentration was estimated using the methylene-blue assay (Cline, 1969). After 5 days and confirmation of active methane oxidation, 1 mM sulfite, 5 mM sulfide, 10 mM thiosulfate, various concentrations of polythionate and polysulfide was added and the rate of methane oxidation was tracked over time. Sulfite concentration was selected based on previous studies on Fsr detoxification (Johnson and Mukhopadhyay, 2005, 2008); sulfide and thiosulfate concentration were selected to show no toxicity effect even at higher concentrations and in line with previous studies on potential metabolic intermediates in AOM (Nauhaus et al., 2005; Wegener et al., 2016); polythionate and polysulfide concentrations were selected to be lower than the previous study (Milucka et al., 2012) in order to demonstrate a metabolic effect even at lowered concentrations.

*Methanococcoides burtonii* was obtained from DSMZ culture collection (DSMZ6242). Cultures were initiated in the DSM280 media, and then transferred to a minimal media without sulfate containing the following ingredients (per 1L media): 0.34 g of KCl, 8.2 g of MgCl_2_.6H_2_O, 0.25 g of NH_4_Cl, 0.014 g of CaCl_2_.2H_2_O, 0.14 g of K_2_HPO_4_, 18 g of NaCl, 5 g of NaHCO_3_, 0.5 g of Na_2_S⋅9H_2_O, vitamin and trace elements solutions as DSM141 except replacing sulfate salts with chloride salts. To study the response of *M. burtonii* to different sulfur compounds, 60 ml of exponentially growing cells were diluted into 90 ml of the media without sulfide, and then 5 ml of the mixture was distributed into Balch tubes anaerobically. Then, an additional 1 mM sulfide was added. The cultures were then flushed briefly and pressurized with 0.15 MPa of N_2_:CO_2_ (80:20) first, then to 0.17 MPa with argon gas. When the cultures reached mid-exponential growth phase, different sulfur compounds from anaerobic stock solutions were added into the cultures in replicates of 4 to the following final concentrations: 0.5 mM of sulfite, 1.0 mM of polythionate, 1.0 mM of polysulfide, 10 mM of thiosulfate, and 5 mM of NaHS. Polythionate and polysulfide solutions were prepared as above. Cultures were incubated at 22°C, and growth was monitored using spectrophotometer at 600 nm.

### Long-Term Incubations With Sulfur Amendments and Community Analysis

We performed long-term incubations amended with different sulfur compounds using sediment ID 5207 from Hydrate Ridge, United States (Supplementary Table 3). This sediment sample was selected based on active methane dependent sulfide production and contained a mixture of ANME lineages. First, the sediment was mixed with 0.22 μm filtered natural bottom seawater collected on cruise AT 18–10 in 1:2 ratio. Then, 10 ml of mixed sediment seawater slurry was aliquoted into 30 ml bottles and capped with black rubber stopper in the anaerobic chamber with a mixed gas atmosphere of N_2_:H_2_ (95:5). 2 ml of mixed slurry was centrifuged at 16,000 × *g* for 30 s and frozen in −20°C for later DNA analysis as the “original” sample. The bottles were then brought out of the anaerobic chamber and flushed with N_2_ for 10 min. Thiosulfate and sulfite were added to a final concentration of 10 mM from 0.22 μm filtered anaerobic stock solutions; polythionate, synthesized as described above, was added to a final concentration of 14 mM from a anaerobic stock solution; sulfur powder, ca. 50 mg steam sterilized overnight, was added to bottles by uncapping the stopper while flushing with N_2_ and quickly recapped. For incubations with CH_4_ headspace, the headspace was flushed for 1 min with CH_4_ then pressurized to 0.250 MPa. The microcosms were mixed and incubated in the dark at 4°C. The overlaying seawater above the sediments was exchanged with the same seawater and amendments every month. Sulfide in the exchanged seawater was first preserved in 0.5 M zinc acetate, and later measured using the methylene-blue assay (Cline, 1969). After 6 months, 0.5 ml of slurry was sampled by centrifuging at 16,000 × *g* for 30 s and immediately flash frozen in liquid nitrogen.

For community analysis, 0.2 g of wet weight sediment were extracted using the PowerSoil DNA Isolation Kit as described above. PCR amplification and barcoding of the 16S rRNA gene were performed as described previously (Case et al., 2015). Sequencing was performed at Laragen, Inc (Culver City, CA, United States) using an Illumina MiSeq platform. Data was analyzed using QIIME 1.8.0 (Caporaso et al., 2010) and processed sequences were assigned to phylotypes using a 99% similarity cutoff to the SILVA database version 115 (Quast et al., 2013) as previously (Case et al., 2015).

## Results and Discussion

### Survey of Sulfur Metabolism in ANME and Methanogen Genomes

Sulfate can be reduced to sulfide for anabolism or catabolism, and distinct assimilatory or dissimilatory pathways have been characterized previously (Verschueren and Wilkinson, 2001; Rabus et al., 2015). Analysis of the genomes from diverse ANME lineages revealed multiple candidate genes for assimilatory but not dissimilatory sulfate reduction (Figure 1). Nitrate-reducing *Ca.* Methanoperedens (formerly known as ANME-2d) recovered from freshwater environments showed a more expanded genetic capacity to reduce sulfate compared to the marine ANME lineages (ANME-1b, ANME-2a, ANME-2b, and ANME-2c) that perform AOM coupled sulfate reduction with deltaproteobacterial partners (Figure 1). This study focuses on the genetic potential of sulfate reduction to sulfide in the marine ANME lineages. The Supplementary Information includes sulfate reduction pathways separated by ANME lineage and a more detailed discussion on *Ca.* Methanoperedens.

**FIGURE 2:**
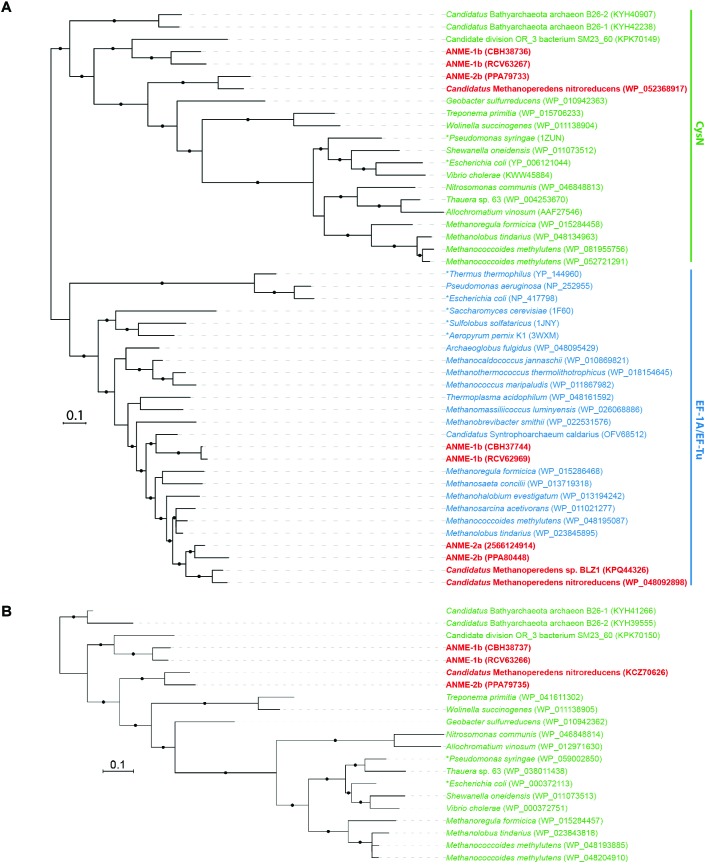
Phylogeny of heterodimeric ATP sulfurylase subunits (CysDN). **(A)** Bayesian phylogeny of 416 amino acid residues of sulfate adenylyltransferase subunit 1 (CysN) and elongation factor 1 alpha (EF-1A) or elongation factor thermo unstable (EF-Tu) proteins. CysN, in green, formed a separate phylogenetic cluster from the homologous EF-1A and EF-Tu in blue. ANME proteins are bolded in red. The phylogenetic analysis distinguished CysN from their homologous elongation factor in ANME. **(B)** Bayesian phylogeny of 270 amino acid residues of sulfate adenylyltransferase subunit 2 (CysD) in green. They are found in ANME genomes next to CysN confirming that they are the heterodimeric ATP sulfurylase subunits. Asterisks (^∗^) indicate proteins that have been studied biochemically or structurally (Liu et al., 1994; Andersen et al., 2000; Vitagliano et al., 2001; Mougous et al., 2006; Schmeing et al., 2009; Kobayashi et al., 2010; Thirup et al., 2015). Protein accession numbers from the NCBI database or gene IDs from the IMG database are shown in parentheses. Black dots on the branches represent Bayesian posterior probability values greater than 90%, and scale bar indicates the number of amino acid substitutions per site.

Putative sulfate transporters were identified in all ANME lineages, but given the substrate promiscuity of these transport systems for different oxyanions (Marietou et al., 2018), the specificity and enzyme activity for sulfate is uncertain. Once sulfate is transported into the cell, the first step in sulfate reduction is the activation of sulfate (sulfur oxidation state +6) using ATP that can be catalyzed by two non-homologous ATP sulfurylase enzymes (Sat or CysDN). The heterodimeric sulfate adenylyltransferase (CysDN) used for sulfate assimilation is composed of a regulatory GTPase subunit CysN and a catalytic subunit CysD, and was previously reported in ANME-1 (Meyerdierks et al., 2010). Our ANME-2b genome also contained a CysDN homolog (Figure 1). CysN and elongation factor 1-alpha (EF-1α) are homologous (Mougous et al., 2006). Phylogenetic analysis confirmed that the CysN in ANME-1 and ANME-2b clustered together with characterized CysN as opposed ot EF-1α (Figure 2A). In addition, the ANME CysN homolog were found next to CysD, which showed a similar evolutionary pattern (Figure 2B and Supplementary Table 1). The CysDN found in ANME would operate at a high energetic cost, requiring one GTP and one ATP per sulfate activated (Liu et al., 1994) and therefore unlikely involved in dissimilatory sulfate reduction. In comparison, only three known methanogens (*Methanoregula formicica*, *Methanococcoides methylutens*, *Methanolobus tindarius*) contained CysDN, which were not monophyletic with the ANME proteins, suggesting that these methanogens may have acquired *cysDN* separately through horizontal gene transfer (Figures 2A,B). The alternative protein for sulfate activation, the homo-oligomeric ATP sulfurylase (Sat), was found in ten methanogens as well as *Ca.* Methanoperedens, but not marine ANME lineages with partner SRB (Supplementary Table 1). Sat is involved in both assimilatory and dissimilatory sulfate reduction and uses one ATP per reaction (Sperling et al., 2001; Ullrich et al., 2001). It is interesting to find CysDN and Sat in a few methanogens and *Ca.* Methanoperedens (see the Supplemental Information for details on sulfur pathway genes in methanogens). Future genetic studies of CysDN and Sat will be needed to confirm their roles in sulfate activation and assimilation in ANME and methanogens.

**FIGURE 3:**
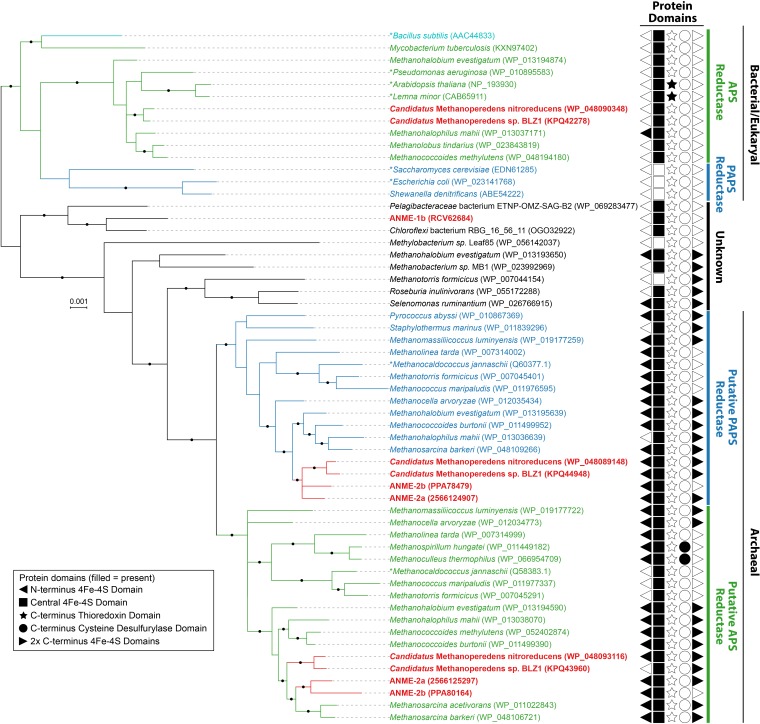
Bayesian phylogeny of assimilatory adenyl-sulfate (APS) reductases and phosphoadenylyl-sulfate (PAPS) reductases. APS reductases and putative APS reductases are in green, PAPS reductases and putative PAPS reductases are in blue, bifunctional APS and PAPS reductase of *Bacillus subtilis* is in teal, and ANME proteins are bolded and in red. Archaeal and Bacterial/Eukaryal sequences formed separate clusters. Asterisks (^∗^) indicate proteins that have been studied biochemically from Archaea (Lee et al., 2011; Cho, 2013), or Bacteria/Eukaryotes (Gutierrez-Marcos et al., 1996; Savage et al., 1997; Suter et al., 2000; Berndt et al., 2004; Kim et al., 2004; Yu et al., 2008). Length of the proteins ranged from 239 to 896 amino acids with the addition of protein domains. The protein domains, if found, are shown with filled symbols. Only 172 amino acid residues of the central shared region were used for phylogenetics. Given that two copies of APS/PAPS reductases were found in each ANME-2 lineage and clustered separately, it is likely one is for APS and the other is for PAPS reduction similar to *M. jannaschii* (Lee et al., 2011; Cho, 2013). *Ca*. Methanoperedens and four other methanogens in Methanosarcinales also contained a second putative assimilatory APS reductase more closely related to the bacterial/eukaryotic homologs, while ANME-1b contained a gene that does not cluster with assimilatory APS or PAPS reductases of known substrate. Protein accession numbers from the NCBI database or gene IDs from the IMG database are shown in parentheses. Black dots on the branches represent Bayesian posterior probability values greater than 90%, and scale bar indicates the number of amino acid substitutions per site.

Activated sulfate in the form of adenosine-5′-phosphosulfate (APS, sulfur oxidation state +6) can be reduced to sulfite (sulfur oxidation state +4) directly through APS reductase, or indirectly via 3′-phosphoadenosine-5′-phosphosulfate (PAPS, sulfur oxidation state +6) that uses APS kinase (CysC) followed by PAPS reductase (Verschueren and Wilkinson, 2001). Genes for dissimilatory APS reductase (AprAB) and the essential membrane complex QmoABC in sulfate reducing bacteria and archaea (Pereira et al., 2011) were not identified in any ANME genomes as reported in previous studies (Meyerdierks et al., 2010; Wang et al., 2014; Krukenberg et al., 2018). We identified APS kinase (*cysC*) in our ANME-1b and ANME-2c genomes (Figure 1), which is in line with previous observations (Meyerdierks et al., 2010; Krukenberg et al., 2018). Previous studies also mentioned the presence of assimilatory APS/PAPS reductase homolog in ANME-1, which we have also identified in ANME-2a and ANME-2b genomes (Figure 1 and Supplementary Table 1). Assimilatory APS reductase and PAPS reductase are homologous and use the same catalytic mechanism (Carroll et al., 2005). These APS/PAPS reductase homologs are also widespread in methanogen genomes (Supplementary Table 1). We further investigated their phylogenetic relationship with characterized homologs, and found a separation between assimilatory APS/PAPS reductases in archaea and those commonly found in bacteria and eukarya (Figure 3). Based on their phylogenetic clustering with biochemically characterized homologs from *Methanocaldococcus jannaschii* (Lee et al., 2011; Cho, 2013), we propose that one cluster is involved in APS reduction while the other cluster is involved in PAPS reduction (Figure 3). Assimilatory APS reductase of *M. jannaschii* is a small protein containing a 4Fe-4S domain (Lee et al., 2011), while the assimilatory PAPS reductase of *M. jannaschii* contains an extra iron-sulfur binding domain at the N-terminus (Cho, 2013). In comparison, homologs from ANME and other methanogen genomes contained additional domains including extra iron-sulfur cluster binding domains at the N- or C-terminus, or a cysteine desulfurylase domain at the C-terminus (Figure 3). Given these sequence differences, we refer to these homologous proteins as putative APS/PAPS reductases. It is possible that the homologs’ enzyme substrate specificity is the same as those in *M. jannaschii*, while the added iron-sulfur clusters could be facilitating electron transfer. The source of APS or PAPS is unclear, as many of the ANME and methanogen genomes lack the genes involved in activating sulfate and phosphorylating APS (Supplementary Table 1).

**FIGURE 4:**
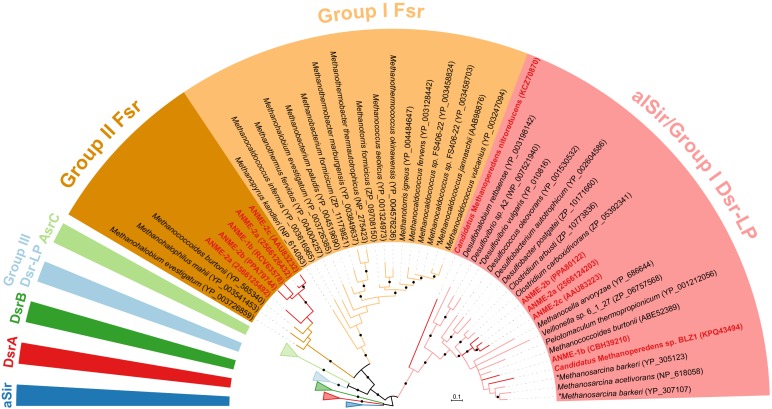
Bayesian phylogeny of sulfite reductases. Two well-supported groups of Fsr were identified in exclusion of alSir and other sulfite reductases. ANME proteins are bolded in red. The phylogenetic tree was constructed based on 224 amino acid residues of the shared catalytic and siroheme-binding region. Asterisks (^∗^) indicate proteins that have been studied biochemically (Huynh et al., 1984; Moura et al., 1986; Johnson and Mukhopadhyay, 2005). Protein accession numbers from the NCBI database or gene IDs from the IMG database are shown in parentheses. Black dots on the branches represent Bayesian posterior probability values greater than 90%, and scale bar indicates the number of amino acid substitutions per site. The fully expanded tree can be found be found in Supplementary Figure 1.

The final step in sulfate reduction involves a reduction of sulfite to sulfide (sulfur oxidation state −2). There are at least seven groups of homologous sulfite reductases that have a proposed assimilatory (aSir, alSir and Fsr) or dissimilatory (DsrA, DsrB, AsrC) function, in addition to a biochemically uncharacterized group Group III Dsr-LP (Dsr-Like Protein) (Dhillon et al., 2005; Loy et al., 2008; Susanti and Mukhopadhyay, 2012). All known dissimilatory sulfite reductases encoding genes were absent from ANME and methanogen genomes (DsrA, DsrB and AsrC, Supplementary Figure 1 and Supplementary Table 1). In addition, genes for the essential membrane complex for dissimilatory sulfate reduction, DsrMK (Pereira et al., 2011), found in all known sulfate-reducing bacteria and archaea were also absent in the ANME genomes investigated. However, all marine ANME lineages with SRB partner contained alSir and Fsr in their genomes (Figures 1, 4), in line with previous ANME genomes (Hallam et al., 2004; Meyerdierks et al., 2010; Wang et al., 2014; Krukenberg et al., 2018). Furthermore, in our phylogenetic analysis of sulfite reductases, it was observed that the previously studied coenzyme F_420_-dependent sulfite reductase (Fsr) from *M. jannaschii* (Johnson and Mukhopadhyay, 2005, 2008) clusters with Fsr genes from other non-cytochrome containing methanogens, here referred to as Group I Fsr. The Fsr homologs in ANME (with the exception of *Ca.* Methanoperedens) and other *Methanosarcinales* genomes formed a distinct well-supported clade, referred to here as Group II Fsr (Figure 4).

To show that Group II Fsr could be found in different methane seep sediments, we designed sets of specific and degenerate PCR primers based on alignments of ANME *fsr* sequences and used them to screen 4 different samples from Hydrate Ridge, United States (Supplementary Table 3). Positive amplicons were recovered from all four samples and the resulting *fsr* sequences clustered with *fsrs* recovered from ANME-2a/2b/2c genomes (Supplementary Figure 5). The ANME-2a reconstructed genome (Wang et al., 2014) has two copies of Group II Fsr, but a primer set designed to specifically target one of the variants (IMG gene ID 2566126432) failed to amplify from our samples.

All Group II Fsr sequences were then analyzed together with alSir and well-characterized DsrA to assess conservation of key amino acid residues. Sulfite reductases in general have conserved amino acid residues involved in the binding of siroheme and sulfite independent of their different physiological roles (Crane et al., 1995; Dhillon et al., 2005; Schiffer et al., 2008). Alignments of both Fsr and alSir showed strong conservation of siroheme-[FeS] binding cysteines also present in DsrA (Supplementary Figure 3). However, the key residues that bind sulfite were changed in the Group II Fsr. Two arginine residues in the sulfite binding site (Crane et al., 1995; Schiffer et al., 2008) were replaced with lysine and glycine in all Group II Fsr sequences (Supplementary Figure 3). This variation was also evident in models of protein homology which showed conservation in the overall structure and 3D positioning of siroheme-[FeS] binding cysteines (Supplementary Figure 4A), but predicted an altered active site pocket due to the replacement of Arg with amino acids Lys or Gly smaller in size (Supplementary Figure 4B). The amino acid changes may suggest a different substrate specificity of Group II Fsr compared to biochemically characterized Group I Fsr.

### Metaproteomic Expression of ANME Assimilatory Sulfur Metabolism Genes

Environmental metaproteomic analysis of methane seep sediments confirmed the active expression of Group II Fsr and other sulfur metabolism genes from ANME (summarized in Table 1, and manual validation of spectra corresponding to these peptides is provided in Supplementary Data Sheet 1). Peptides assigned to CysN, APS kinase and a putative APS/PAPS reductase homolog associated ANME-1 were detected (Table 1), suggesting that ANME-1 may be actively assimilating sulfate in the environment. Assimilation of sulfate would be particularly beneficial for ANME-1 at the base of or below the sulfate-methane transition zone where sulfate levels are low (Beulig et al., 2018). In contrast, the only detected proteins closely affiliated with ANME-2a and ANME-2b were two sulfite reductases, alSir and Group II Fsr, and a putative sulfate transporter (Table 1).

**Table 1:** Specific search for sulfur pathway proteins of marine ANME lineages in methane seep metaproteomes.

Protein accession	Description	Organism	Averaged normalized spectral counts (nSpc) in methane seep metaproteomes				
			Hydrate ridge	Santa monica 0–4 cm	Santa monica 8–12 cm	Eel river 0–10 cm	Eel river 10–20 cm
RCV62684	Unknown APS/PAPS reductase	ANME-1b	b.d.	b.d.	b.d.	b.d.	71.5
RCV63267	CysN	ANME-1b	b.d.	484.4	b.d.	b.d.	b.d.
RCV64987	SulP family inorganic anion permease	ANME-1b	b.d.	b.d.	b.d.	17.7	b.d.
CBH38748	APS Kinase	ANME-1b	1562.6	b.d.	b.d.	b.d.	b.d.
2566123967	DASS family sodium-coupled anion symporter	ANME-2a	b.d.	b.d.	b.d.	103.6	b.d.
PPA79744	Group II Fsr	ANME-2b	2490.6	b.d.	b.d.	b.d.	b.d.
PPA80122	alSir	ANME-2b	4762.7	b.d.	b.d.	b.d.	b.d.
AAU83232	Group II Fsr	ANME-2c	3964.9	b.d.	1243.2	b.d.	19
AAU83223	alSir	ANME-2c	5775.2	1684	b.d.	b.d.	b.d.
MH823235	Group II Fsr	Unknown ANME	6395.9	b.d.	1824.2	b.d.	38
MH823238	Group II Fsr	Unknown ANME	1215.6	b.d.	b.d.	b.d.	b.d.

*The search used a streamlined database containing only sulfur proteins of interest, and peptide fragmentation spectra of proteins found to be expressed were also manually validated in Supplementary Data Sheet 1. b.d., below detection limits of mass spectrometry.*

Of all the ANME sulfur pathway proteins recovered, alSir and Fsr had the highest relative expression levels (Table 1). However, expression was at least 10-fold below the relative expression of methane oxidation genes and the dissimilatory sulfate reduction genes present in the syntrophic SRB partner (Supplementary Table 2). This result is similar to findings in a recent metatranscriptomic study of AOM enrichments (Krukenberg et al., 2018), and appears inconsistent with a role in energy generating, dissimilatory functions, such as sulfate reduction to zero-valent sulfur (Milucka et al., 2012). In our genomic survey of ANME and methanogens, *alSir* was more widespread than *fsr* and most of the *alSir*-containing species did not have the full assimilatory sulfate reduction pathway (Supplementary Table 1). The physiological role of alSir could be sulfite assimilation, but a source for *in situ* sulfite production remains unclear. Another possible role of alSir could be intracellular production of the essential sulfite for coenzyme M biosynthesis (Graham et al., 2009) by the reverse reaction (oxidizing sulfide to sulfite) as previously proposed (Moura et al., 1982). Given the high levels of *in situ* protein expression of Group II Fsr by ANME-2 (Table 1) and change in their active site residues (Supplementary Figure 3), further biochemical investigation are needed to confirm the enzyme substrate and reaction.

### Metabolic Response of ANME and *Methanococcoides burtonii* to Sulfite and Zero-Valent Sulfur

**FIGURE 5:**
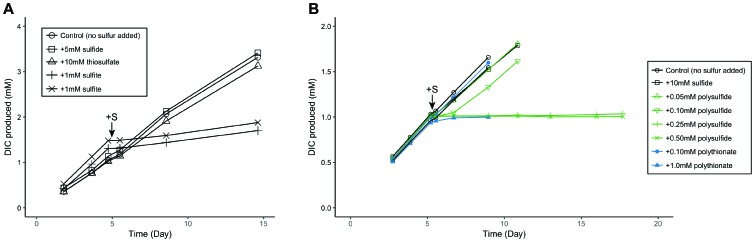
Metabolic response of ANME to **(A)** sulfite and **(B)** zero-valent sulfur additions, as measured by ^13^C-labeled dissolved inorganic carbon (DIC) production from ^13^CH_4_. Zero-valent sulfur was added in the form of polysulfide and polythionate. Arrows indicate time of sulfur compound additions. Sulfite (1 mM) and zero-valent sulfur (>0.25 mM polysulfide or 1.0 mM polythionate) additions showed an inhibitory effect on methane oxidation in contrast to control or other sulfur compounds. Methane seep sediment ID 7142, dominated by ANME-2a and ANME-2c lineages, was used in these experiments.

To explore the potential roles of these sulfite reductases in ANME, we conducted microcosm experiments using a methane seep sediment (sediment ID 7142, dominated by ANME-2a/2c) amended with sulfite. Given Group I Fsr’s potential sulfite detoxification role in *M. jannaschii* (Johnson and Mukhopadhyay, 2008), we hypothesize that Group II Fsr may also function in sulfite detoxification. Addition of sulfite at concentration of 1.0 mM was found to be inhibitory to ANME, leading to an immediate decrease in the rate of AOM (Figure 5A). *Methanococcoides burtonii*, a close relative of ANME-2 within the *Methanosarcinales*, also contains alSir and Group II Fsr (Figure 4). Similar to ANME experiments, sulfite was also found to be inhibitory to the growth of *M. burtonii*, as observed by optical density measurements of the cultures (Supplementary Figure 2). These results contrast previous publications showing the effect of Group I Fsr on sulfite tolerance, where heterologous expression of Group I Fsr of *M. jannaschii* resulted in growth of *Methanococcus maripaludis* with 2 mM sulfite (Johnson and Mukhopadhyay, 2008). Although these experiments were conducted with different methanogens, there seems to be a difference in sulfite tolerance or maybe function between Group I and Group II Fsr.

Zero-valent sulfur has been proposed as a metabolic intermediate in the AOM symbiosis (Milucka et al., 2012). We used microcosm experiments to investigate the effect of zero-valent sulfur on ANME activity. An inhibitory effect of zero-valent sulfur in the forms of polythionate and polysulfide at concentrations of 1.0 and 0.25 mM, respectively, was observed on methane oxidation (Figure 5B). Following thermodynamic predictions by Milucka et al. (2012), product inhibition on methane oxidation by zero-valent sulfur should only occur at much higher concentrations (ΔG’ = 0 when [HS_2_^-^] = 6193 M), assuming ANME directly coupled methane oxidation to dissimilatory sulfate reduction producing zero-valent sulfur in the form of disulfide. The effect of zero-valent sulfur on AOM measured in our experiments is therefore unlikely due to product inhibition but an alternative toxic mechanism unknown at the moment. *M. burtonii*, a closely related methanogenic archaeon to ANME-2, also stopped growing upon addition of 1 mM polysulfide (Supplementary Figure 2), supporting that zero-valent sulfur is toxic to this phylogenetic group rather than specifically to ANME. Furthermore, we could not enrich for the partner SRB in methane seep microcosms amended with polythionate or polysulfide (Supplementary Figure 6). This similar finding has been reported previously (Wegener et al., 2016). Combined, these results indicate that zero-valent sulfur is unlikely a metabolic intermediate in the AOM symbiosis.

### Ecological Relevance of Assimilatory Sulfate Reduction Genes in ANME

By recovering new ANME genomes and surveying their sulfur pathways, our results revealed the genomic potential for several ANME lineages to assimilate sulfur species more oxidized than sulfide. There are predicted differences between major ANME lineages in both sulfate activation by heterodimeric ATP sulfurylases (CysDN) found in ANME-1/2a and *Ca.* Methanoperedens, and the formation of sulfite using assimilatory APS/PAPS reductases found in ANME-2a/2b and *Ca.* Methanoperedens (Figure 1). Two sulfite reductases, alSir and Group II Fsr, were found to be the highest expressed proteins in methane seep sediment related to sulfur cycling in ANME (Table 1). However, their expression levels were still much lower than that of primary metabolisms, i.e., methane oxidation in ANME and dissimilatory sulfate reduction in SRB. Together with information on their characterized homologs associated with assimilatory but not dissimilatory sulfate reduction, our results suggest that ANME are unlikely to perform dissimilatory sulfate reduction as proposed previously (Milucka et al., 2012). Additional experiments are needed to determine the enzyme function of two sulfite reductases that are common to all marine ANME lineages, as well as the divergent homologs of ATP sulfurylase and assimilatory APS/PAPS reductases that were found in some ANME lineages. These genes may be important for the synthesis of essential organo-sulfur molecules, in particular coenzyme M that has a sulfonate group at +4 oxidation state. The differences in sulfur assimilatory genes between ANME lineages, representing novel order to genus-level diversity, underscore the phylogenetic as well as physiological differences between them (see Supplementary Information for a more detailed discussion).

It is intriguing to find potential genes for assimilation of sulfate or other sulfur species more oxidized than sulfide in ANME genomes, especially ANME-1b/2a/2b lineages that live in syntrophy with SRB partners and high levels of sulfide. In marine sediments with active sulfur cycling, such as sulfate-methane transition zones where ANME thrive, sulfate and sulfide may not be the only sulfur species present. Sulfite and thiosulfate have previously been measured at low micromolar concentrations in different marine sediments including methane seep sediment (Zopfi et al., 2004; Smith et al., 2017). Under these conditions, the ability to scavenge additional sulfur species for anabolism could be beneficial. In addition, ANME-1b and ANME-2a/2b/2c lineages have been found together with microorganisms other than deltaproteobacterial sulfate reducers that hints alternative syntrophic lifestyles (Hatzenpichler et al., 2016), and ANME-2a/2c remained anabolically and catabolically active in laboratory incubations devoid of sulfate using electron acceptors including AQDS, humic acids and Fe^(III)^ (Scheller et al., 2016). In these scenarios, the ability to assimilate multiple sulfur sources using Group II Fsr or other enzymes in the assimilatory sulfate reduction pathway may provide ANME, or methane-cycling archaea in general, a broader environmental niche and the ability to survive in environments with different anabolic sources of sulfur.

## Data Availability

The ANME genomes generated for this study have been deposited at NCBI GenBank database under the Whole Genome Shotgun project accession numbers QENH00000000, MZXQ00000000, and PYCL00000000 for ANME-1b (ANME sp. CONS3730B06UFb1), ANME-2b (ANME sp. HR1), and ANME-2c (ANME sp. S7142MS2) respectively. Protein sequences and alignments analyzed for this study can be found on FigShare: 10.6084/m9.figshare.7035917, 10.6084/m9.figshare.7036289, and 10.6084/m9.figshare.7037228.

## Author Contributions

HY, RH, BM, and VO designed research. HY, DS, SM, CS, KC, RI, SS, and PT performed research and data analysis. HY and VO wrote the paper with contribution from all authors.

## Conflict of Interest Statement

The authors declare that the research was conducted in the absence of any commercial or financial relationships that could be construed as a potential conflict of interest.

## Footnotes

1 https://sourceforge.net/projects/bbmap/

2 http://sourceforge.net/projects/bbmap/

3 https://github.com/wwood/finishm

4 https://github.com/dparks1134/RefineM
