# Supplementary figures and images for: Comparative Genomics and Proteomic Analysis of Assimilatory Sulfate Reduction Pathways in Anaerobic Methanotrophic Archaea
Source: Front Microbiol. 2018 Dec 3;9:2917. doi: 10.3389/fmicb.2018.02917 (PMC6286981; doi:10.3389/fmicb.2018.02917)

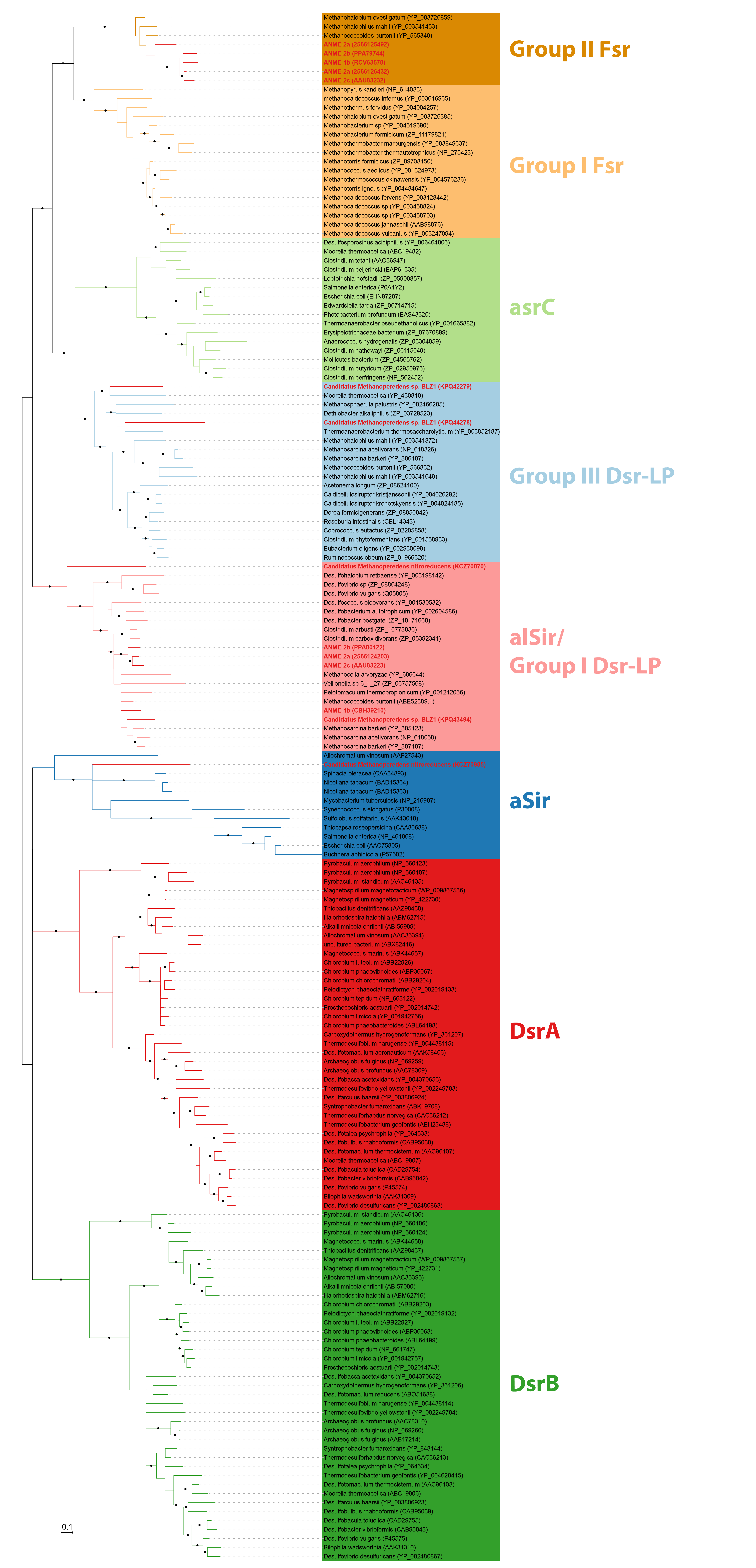

Supplement: Supplementary file 1 [file Image_1.JPEG]
