# Supplementary material for: Comparative Genomics and Proteomic Analysis of Assimilatory Sulfate Reduction Pathways in Anaerobic Methanotrophic Archaea
Source: Front Microbiol. 2018 Dec 3;9:2917. doi: 10.3389/fmicb.2018.02917 (PMC6286981; doi:10.3389/fmicb.2018.02917)
Supplement: Supplementary file 4 [file Data_Sheet_1.pdf]

Supplementary Material

Comparative Genomics and Proteomic Analysis of Assimilatory Sulfate Reduction Pathways in Anaerobic Methanotrophic Archaea

Hang Yu[1,2], Dwi Susanti[3], Shawn E. McGlynn[1,8], Connor T. Skennerton[1], Karuna Chourey[4], Ramsunder Iyer[4,5], Silvan Scheller[1,9], Patricia L. Tavormina[1], Robert L. Hettich[4], Biswarup Mukhopadhyay[3,6,7], Victoria J. Orphan[1*]

[1]Division of Geological and Planetary Sciences, California Institute of Technology, Pasadena, CA 91125, USA

[2]Ronald and Maxine Linde Center for Global Environmental Science, California Institute of Technology, Pasadena, CA 91125, USA

[3]Department of Biochemistry, Virginia Tech, Blacksburg, VA 24061, USA

[4]Chemical Sciences Division, Oak Ridge National Laboratory, Oak Ridge, TN 37831, USA

[5]Graduate School of Genome Science and Technology, University of Tennessee, Knoxville, Knoxville, TN 37996, USA

[6]Biocomplexity Institute, Virginia Tech, Blacksburg, VA 24061, USA

[7]Virginia Tech Carilion School of Medicine, Virginia Tech, Blacksburg, VA 24061, USA

[8]Present Address: Earth-Life Science Institute, Tokyo Institute of Technology, Ookayama, Meguro-ku, Tokyo, 152-8550, Japan

[9]Present Address: Department of Bioproducts and Biosystems, Aalto University, Kemistintie 1, 02150 Espoo, Finland

*** Correspondence:**
Victoria J. Orphan
vorphan@gps.caltech.edu

Keywords: sulfur pathway, sulfate reduction, anaerobic oxidation of methane, ANME, syntrophy.

1 Supplementary Information

1.1 Sulfur assimilation genes in different ANME lineages

ANME represent diverse phylogenetic and physiological lineages in the class *Methanomicrobia* that carry out AOM with different electron acceptors or syntrophic partners (Knittel and Boetius, 2009). It was unexpected to find assimilatory sulfate reduction genes in ANME genomes (Figure 1), especially within ANME-1 and ANME-2a/2b/2c lineages that live in symbiosis with sulfate-reducing bacteria in sulfidic environments. Related methanogenic archaea could also be found in sulfidic environments, and all cultured methanogens have accordingly been shown to derive anabolic sulfur from sulfide (Liu et al., 2012). Here we discuss the sulfur assimilation genes found in each ANME lineage highlighting the differences.

The ANME-2a genomes contained assimilatory APS and PAPS reductases (Figure 3), alSir and two different Group II *fsr* (Figure 4). However, we did not identify any known sulfate adenylyltransferases, either *cysDN* or *sat*, that could activate sulfate. In comparison, the sulfur pathways in ANME-2b genome were similar to that of ANME-2a, except heterodimeric ATP sulfurylases (*cysDN*) were found in ANME-2b. Together ANME-2b could have a complete pathway to reduce sulfate to sulfide with APS and sulfite as the intermediates for assimilation. It might also be possible for these to be used exclusively for sulfite production. Given the need of sulfite by ANME and methanogens to synthesize coenzyme M (Graham et al., 2009), the sulfur genes identified here may represent two different ways to generate sulfite: 1) reduction of sulfate using CysDN and putative APS reductase, and 2) oxidation of sulfide using alSir, which has been hypothesized previously (Moura et al., 1982; White, 1986). The presence of a putative PAPS reductase in both ANME-2a and ANME-2b genomes is puzzling because APS kinase that produces its substrate (namely PAPS) was not found in the genome bins. Our study inferred the function based on phylogenetics (Figure 3) and previous characterized version in *M. jannaschii* (Cho, 2013). The enzyme function of assimilatory PAPS reductase, as well as assimilatory APS reductase, needs to be confirmed biochemically for *Methanosarcinales* including ANME-2.

ANME-2c genomes revealed a different sulfur gene repertoire compared to ANME-2a and ANME-2b. Sulfite reductases, namely alSir and Group II *fsr*, were shared between ANME-2a/2b/2c. However, no assimilatory APS or PAPS reductases have been found in our ANME-2c genome survey. This is puzzling since ANME-2c genome contained *cysDN* and APS kinase (*cysC*) to activate sulfate and form PAPS. It maybe that PAPS is used for assimilatory purposes such as sulfation reactions (Leustek et al., 2000). The presence of sulfite reductases alone without potential pathways to produce sulfite suggests the possibility for a source of sulfite from the environment.

ANME-1b, which is phylogenetically distant from ANME-2a/2b/2c and *Ca.* Methanoperedens, had similar sulfate assimilation genes compared to ANME-2c. A distant homolog of assimilatory APS/PAPS reductase were identified in the ANME-1b genome, but is too distantly related to those in other archaea or bacteria to infer its function based on phylogenetics (Figure 3). ANME-1b also possess rhodanese-like proteins (or thiosulfate sulfurtransferase, *tst*), and might be used to assimilate thiosulfate. Based on the presence of rhodanese or other protein domains, *tst* can be classified as single-domain (Ga0123266_104918, KCZ72772.1, KPQ43738.1), tandem-domain (CBH36927.1, CBH36931.1, CBH36927.1, CBH37402.1, KCZ72976.1), or multi-domain proteins (Ga0123266_10257, Ga0123266_11066, KCZ71040.1, KPQ45278.1) (Cipollone et al., 2007). This protein superfamily transfers a thiol group from thiosulfate or possibly polysulfide to a range of nucleophilic acceptors with potential physiological roles varying from cyanide detoxification, cysteine and iron-sulfur protein cofactors synthesis, and sulfur transport (Aussignargues et al., 2012;

Cipollone et al., 2007; Westley, 1973). Previous work suggested that thiosulfate might serve as electron acceptor for some groups of ANME-1 (Jagersma et al., 2012), but the exact role of *tst* in ANME remains to be elucidated. More recent study of a thermophilic ANME-1 enrichment showed that this lineage could not grow using thiosulfate as the electron acceptor for methane oxidation (Wegener et al., 2016).

Ca. Methanoperedens possesses additional genes in sulfur assimilation that is different in the two genomes investigated here, *Ca.* M. nitroreducens and *Ca.* Methanoperedens sp. BLZ1. Besides CysDN, putative APS reductase, and alSir like in ANME-2a and ANME-2b lineages, *Ca.* M. nitroreducens had a complete assimilatory sulfate reduction pathway including the homo-oligomeric ATP sulfurylase (Sat), bacterial APS reductases, and aSir. These are the canonical sulfate assimilatory genes in some bacteria and plants (See below for more details). This pathway was not identified in *Ca.* Methanoperedens sp. BLZ1, which has a Group I Dsr-LP instead. It is intriguing for *Ca.* Methanoperedens to possess more than one sulfur assimilation genes that catalyze the same reaction. In our metaproteome analysis, CysN, APS reductase and a putative APS/PAPS reductase homologs associated with *Ca.* Methanoperedens and ANME-1b were detected (Supplementary Table 5). In addition, both aSir and Group III Dsr-LP exclusively associated with *Ca.* Methanoperedens were detected (Supplementary Table 5). *Ca.* Methanoperedens genomes used in our proteome searches are of freshwater origin, and the homologs detected based on unique peptide sequences in our analysis were more likely associated with their related marine clade GoM-ArcI as identified by 16S rRNA gene surveys. No methane-oxidizing GoM-ArcI genome is currently available. Previous bioreactor studies confirmed that *Ca.* M. nitroreducens could grow with sulfate as the sole sulfur source in the media (Arshad et al., 2015; Haroon et al., 2013). Furthermore, all the sulfur assimilation genes in *Ca.* M. nitroreducens were found to be expressed in the transcriptome (Haroon et al., 2013).

The observed differences in sulfur pathways of ANME groups is substantial, and may be explained by their phylogenetic and ecological differences. This result may hint that ANME and methanogens could thrive in environments depleted in sulfide, from other sulfur sources such as thiosulfate or maybe even sulfate to generate sulfide for anabolism. Alternatively, the different sulfur assimilation genes are used oxidatively or reductively to generate sulfite needed for a key molecule in methane metabolism, coenzyme M.

1.2 Sulfur assimilation genes in methanogens that use sulfur sources other than sulfide

Previous culturing studies have explored sulfur utilization capability of different methanogens, and below we summarize potential sulfur assimilation genes in their genomes.

Two methanogens, namely *Methanothermococcus thermolithotrophicus* and *Methanobrevibacter ruminantium*, have been reported to use sulfate as the sole sulfur source (Daniels et al., 1986; Rajagopal and Daniels, 1986), and neither contained *cysDN* in their genomes. Instead, *M. thermolithotrophicus* contained the gene encoding Sat, similar to that in the methanotrophic *Ca.* Methanoperedens. While Sat was also detected in ten other methanogens belonging to the orders *Methanocellales*, *Methanococcales* and *Methanosarcinales* (Supplementary Table 1), it was absent in *M. ruminantium*, the growth of which is dependent on sulfur-containing coenzyme M and yeast extract (with sulfur containing amino acids) (Rajagopal and Daniels, 1986; Taylor et al., 1974). The exclusive growth on sulfate in this methanogen and utilization of Sat in others, needs to be tested further to demonstrate their physiological function.

On the other hand, methanogens in the orders of *Methanobacteriales*, *Methanococcales* and *Methanosarcinales* have been reported to grow with sulfur compounds of intermediate oxidation states including elemental sulfur, thiosulfate, or sulfite as their sole sulfur source (Daniels et al.,

1986; Rajagopal and Daniels, 1986) (For exact species and their sulfur use, see notes in Supplementary Table 1). Also, several methanogen orders including *Methanobacteriales*, *Methanococcales*, *Methanomicrobiales* and *Methanosarcinales* have also been found to perform sulfur reduction decoupled to growth, but the genetic mechanism was not described (Stetter and Gaag, 1983). Seven out of 88 representative methanogen genomes were found to have an assimilatory APS reductase gene that is phylogenetically related to bacteria (Figure 3), and all of them also have ATP sulfurylase required for activating sulfate (Supplementary Table 1). In addition, putative APS/PAPS reductases found in ANME were also common in many methanogens (Supplementary Table 1). Homologs of these in other methanogens also occurred with two iron-sulfur binding domains at the C-terminus. Additionally, a few were found to have a cysteine desulfurylase domain at the C-terminus (Figure 3). Most genomes also contain two copies of the putative APS/PAPS reductases, possibly specialized for either APS or PAPS reduction. Furthermore, *M. thermolithotrophicus* that was reported to reduce sulfate also contain homologs of dissimilatory APS reductase genes (*aprAB*). Despite the prevalence of putative APS/PAPS reductases in methanogenic and methanotrophic archaeal genomes, it is particular intriguing that the source of their substrates (APS or PAPS) is unclear, since many of the genomes do not have homologs of ATP sulfurylase or APS kinase (Supplementary Table 1).

None of the methanogen genomes contained dissimilatory sulfite reductase (*dsrAB*), including *M. thermolithotrophicus* with *sat* and *aprAB* (Supplementary Table 1). Also *M. thermolithotrophicus* lacks the essential membrane complex, encoded by *dsrMK*, for energy conservation. It is therefore likely that *M. thermolithotrophicus* cannot perform dissimilatory sulfate reduction. aSir that was found in *Ca.* M. nitroreducens, was only found in two other methanogens, while Dsr-like proteins (alSir/Group I Dsr-LP and Group III Dsr-LP) are found in a number of methanogens (Supplementary Table 1) (Susanti and Mukhopadhyay, 2012). The physiological function of these Dsr-like proteins is unknown currently. Overall, the focused comparative genomic analysis of both methanogenic and methanotrophic euryarchaeota suggest few could reduce sulfate directly, but, assimilation of sulfur species of intermediate oxidation states could be possible with putative APS/PAPS reductases and sulfite reductases.

1.3 Assimilatory sulfate reduction pathway in bacteria and eukarya

In bacteria such as *Escherichia coli* and *Salmonella typhimurium*, the assimilatory sulfate reduction pathway proceeds through heterodimeric ATP sulfurylase (*cysDN*), APS kinase (*cysC*), PAPS reductase (*cysH*) and assimilatory sulfite reductase (*alSir* or *cysI*) (Verschueren and Wilkinson, 2001). In anoxygenic phototrophic bacteria, a dedicated sulfate assimilatory pathway exist and skips PAPS as the intermediate, using *cysDN* or *sat*, APS reductase and aSir (Frigaard and Dahl, 2008; Neumann et al., 2000). In yeast, the assimilatory pathway is similar to that in *E. coli*, except *cysDN* has been swapped with the homo-oligomeric ATP sulfurylase *sat* (Thomas and Surdin-Kerjan, 1997; Ullrich et al., 2001). In plants, the assimilatory sulfate reduction pathway is similar to that in anoxygenic phototrophic bacteria skipping PAPS and involves sat, assimilatory APS reductase and aSir (also called ferredoxin-dependent sulfite reductase), although PAPS molecules are still produced from APS using APS kinase for sulfation reactions (Leustek et al., 2000).

1.4 References for Supplementary Information

Arshad, A., Speth, D. R., de Graaf, R. M., Op den Camp, H. J. M., Jetten, M. S. M., and Welte, C. U. (2015). A metagenomics-based metabolic model of nitrate-dependent anaerobic oxidation of

methane by methanoperedens-like archaea. *Front Microbiol* 6, 1423. doi:10.3389/fmicb.2015.01423.

Aussignargues, C. C., Giuliani, M.-C. M., Infossi, P. P., Lojou, E. E., Guiral, M. M., Giudici-Orticoni, M.-T. M., et al. (2012). Rhodanese functions as sulfur supplier for key enzymes in sulfur energy metabolism. *J. Biol. Chem.* 287, 19936–19948. doi:10.1074/jbc.M111.324863.

Cho, M. K. (2013). Discovery of novel 3'-phosphoadenosine-5'-phosphosulfate (PAPS) reductase from methanarcheon *Methanocaldococcus jannaschii*.

Cipollone, R. R., Ascenzi, P. P., and Visca, P. P. (2007). Common themes and variations in the rhodanese superfamily. *IUBMB Life* 59, 51–59. doi:10.1080/15216540701206859.

Daniels, L., Belay, N., and Rajagopal, B. S. (1986). Assimilatory reduction of sulfate and sulfite by methanogenic bacteria. *Appl Environ Microbiol* 51, 703–709.

Frigaard, N.-U., and Dahl, C. (2008). “Sulfur Metabolism in Phototrophic Sulfur Bacteria,” in Advances in Microbial Physiology. (Elsevier), 103–200. doi:10.1016/S0065-2911(08)00002-7.

Graham, D. E., Taylor, S. M., Wolf, R. Z., and Namboori, S. C. (2009). Convergent evolution of coenzyme M biosynthesis in the Methanosarcinales: cysteate synthase evolved from an ancestral threonine synthase. *Biochem. J.* 424, 467–478. doi:10.1042/BJ20090999.

Haroon, M. F., Hu, S., Shi, Y., Imelfort, M., Keller, J., Hugenholtz, P., et al. (2013). Anaerobic oxidation of methane coupled to nitrate reduction in a novel archaeal lineage. *Nature* 500, 567–570. doi:10.1038/nature12375.

Jagersma, C. G., Meulepas, R. J. W., Timmers, P. H. A., Szperl, A., Lens, P. N. L., and Stams, A. J. M. (2012). Enrichment of ANME-1 from Eckernförde Bay sediment on thiosulfate, methane and short-chain fatty acids. *Journal of Biotechnology* 157, 482–489. doi:10.1016/j.jbiotec.2011.10.012.

Knittel, K., and Boetius, A. (2009). Anaerobic oxidation of methane: progress with an unknown process. *Annu Rev Microbiol* 63, 311–334. doi:10.1146/annurev.micro.61.080706.093130.

Leustek, T., Martin, M. N., Bick, J. A., and Davies, J. P. (2000). Pathways and regulation of sulfur metabolism revealed through molecular and genetic studies. *Annu. Rev. Plant Physiol. Plant Mol. Biol.* 51, 141–165. doi:10.1146/annurev.arplant.51.1.141.

Liu, Y., Beer, L. L., and Whitman, W. B. (2012). Methanogens: a window into ancient sulfur metabolism. *Trends Microbiol* 20, 251–258. doi:10.1016/j.tim.2012.02.002.

Moura, J., Moura, I., Santos, H., Xavier, A. V., Scandellari, M., and LeGall, J. (1982). Isolation of P590 from *Methanosarcina barkeri* - evidence for the presence of sulfite reductase activity. *Biochem. Biophys. Res. Commun.* 108, 1002–1009.

Neumann, S., Wynen, A., Trüper, H. G., and Dahl, C. (2000). Characterization of the *cys* gene locus from *Allochromatium vinosum* indicates an unusual sulfate assimilation pathway. *Mol. Biol. Rep.* 27, 27–33.

Rajagopal, B. S., and Daniels, L. (1986). Investigation of mercaptans, organic sulfides, and inorganic sulfur-compounds as sulfur sources for the growth of methanogenic bacteria. *Current Microbiology* 14, 137–144.

Stetter, K. O., and Gaag, G. (1983). Reduction of molecular sulphur by methanogenic bacteria. 305, 309–311. doi:10.1038/305309a0.

Susanti, D., and Mukhopadhyay, B. (2012). An intertwined evolutionary history of methanogenic archaea and sulfate reduction. 7, e45313. doi:10.1371/journal.pone.0045313.

Taylor, C. D., McBride, B. C., Wolfe, R. S., and Bryant, M. P. (1974). Coenzyme M, essential for growth of a rumen strain of *Methanobacterium ruminantium*. *J Bacteriol* 120, 974–975.

Thomas, D., and Surdin-Kerjan, Y. (1997). Metabolism of sulfur amino acids in *Saccharomyces cerevisiae*. *Microbiol. Mol. Biol. Rev.* 61, 503–532.

Ullrich, T. C., Blaesse, M., and Huber, R. (2001). Crystal structure of ATP sulfurylase from *Saccharomyces cerevisiae*, a key enzyme in sulfate activation. *EMBO J.* 20, 316–329. doi:10.1093/emboj/20.3.316.

Verschueren, K. H., and Wilkinson, A. J. (2001). *Sulfide: Biosynthesis from Sulfate*. Chichester, UK: John Wiley & Sons, Ltd doi:10.1038/npg.els.0001405.

Wegener, G., Krukenberg, V., Ruff, S. E., Kellermann, M. Y., and Knittel, K. (2016). Metabolic capabilities of microorganisms involved in and associated with the anaerobic oxidation of methane. *Front Microbiol* 7, 869. doi:10.3389/fmicb.2016.00046.

Westley, J. (1973). "Rhodanese," in *Advances in Enzymology and Related Areas of Molecular Biology* Meister/Advances. ed.A. Meister (Hoboken, NJ, USA: John Wiley & Sons, Inc.), 327–368. doi:10.1002/9780470122846.ch5.

White, R. H. (1986). Intermediates in the biosynthesis of coenzyme M (2-mercaptoethanesulfonic acid). *Biochemistry* 25, 5304–5308. doi:10.1021/bi00366a047.

2 Supplementary Figures and Tables

2.1 Supplementary Figures

Supplementary Figure 1. Bayesian phylogeny of sulfite reductases. ANME proteins are bolded and in red. The phylogenetic tree was constructed based on 224 amino acid residues of the shared catalytic and siroheme-binding region. Protein accession numbers from the NCBI database or gene IDs from the IMG database are shown in parentheses. Black dots on the branches represent Bayesian posterior probability values greater than 90%, and scale bar indicates the number of amino acid

substitutions per site. In addition to Fsr and alSir, aSir and Group III Dsr-LP were identified in *Ca.* Methanoperedens. (Attached separatedly)

Supplementary Figure 2. Responses of *M. burtonii* to addition of sulfur compounds. Arrow indicates time of sulfur compound additions. Sulfite and polysulfide addition showed an immediate cessation in the growth of *M. burtonii* in contrast to thiosulfate, sulfate or control without addition.

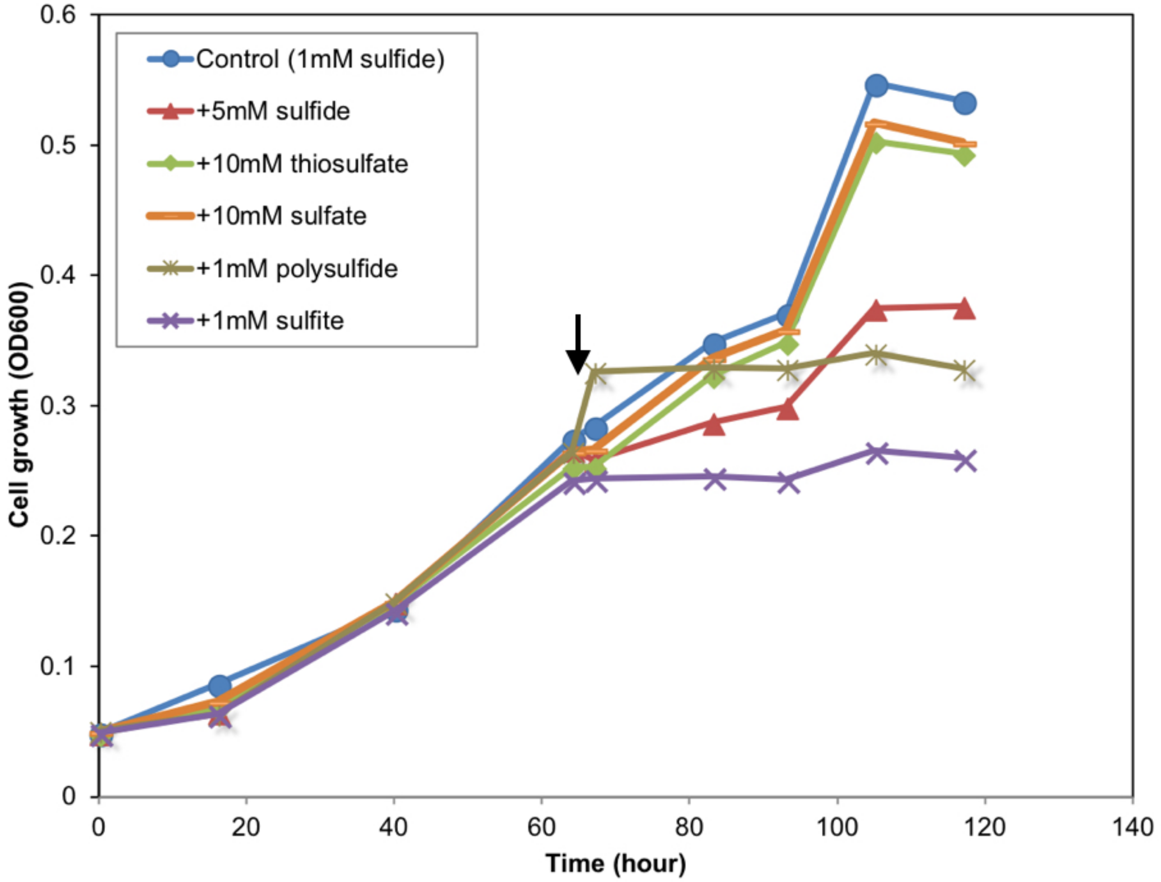

Supplementary Figure 3. Comparison of the protein sequence alignments between alSir, Group I/II Fsr and DsrA. Highly conserved residues in the alignment are highlighted by blue. Four key residues that bind sulfite are indicated by green dots above. The first sulfite-binding Arg in alSir was not conserved in the alignment and might be shifted. DsrA and Group I Fsr showed conservation of all sulfite-binding residues, whereas Group II Fsr showed changes in the two sulfite-binding Arg. The residues in siroheme-binding motif ($CX_5CX_nCX_3C$) and one of the iron sulfur motifs ($CX_2CX_2CX_3C$) are indicated by orange and black dots above, respectively. Arrows indicate the sulfite- and siroheme-binding region used for phylogenetic analysis. Only a portion of the sequences and the

alignment is shown here. The full alignment that includes all sequences in Supplementary Figure 1 can be found on FigShare 10.6084/m9.figshare.7037228.

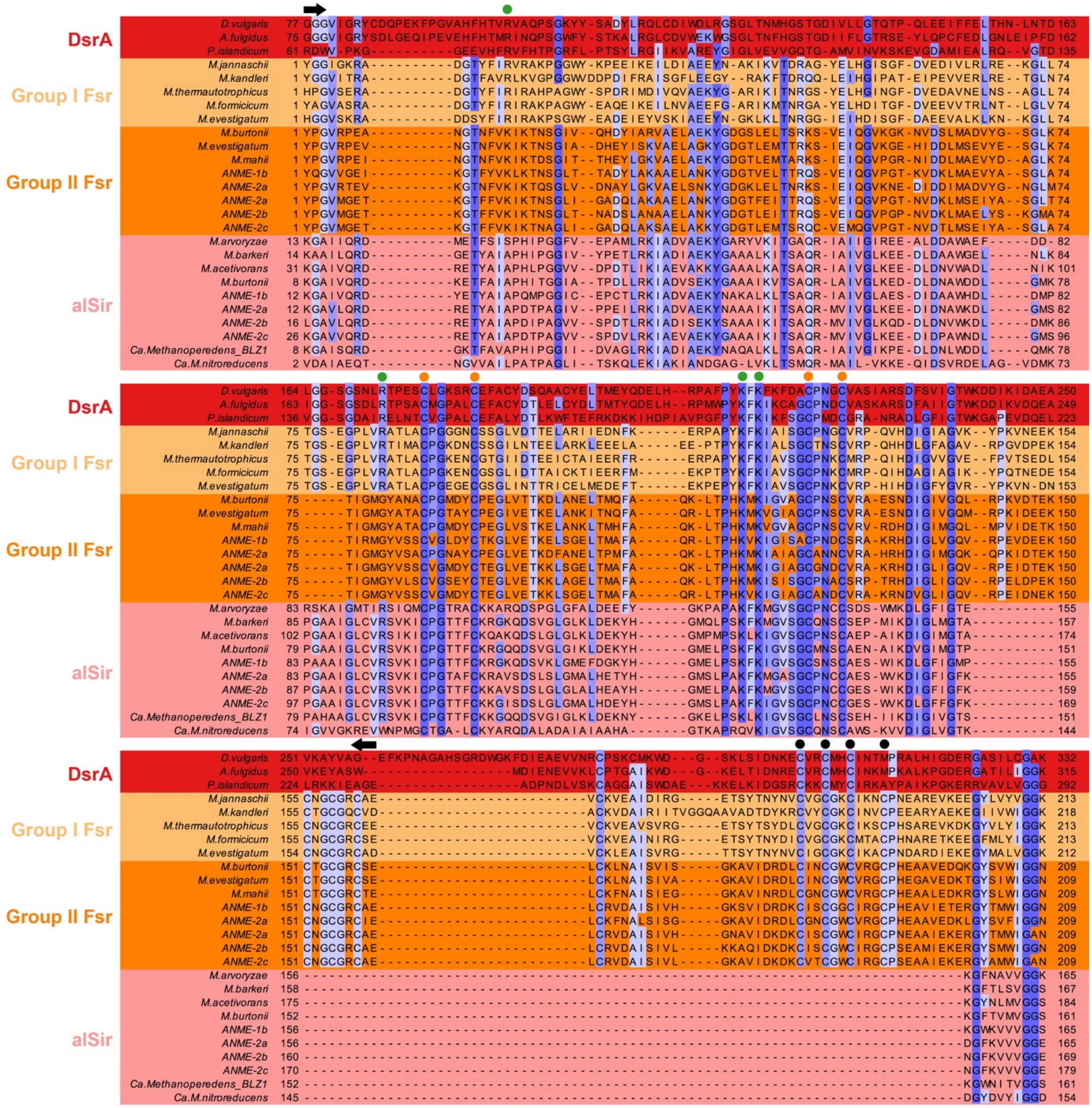

Supplementary Figure 4. Protein homology modeling of the C-terminus domain of Group II Fsr. (**a**) The overall 3D structural similarity between C-terminus of Group II Fsr of ANME-2a (IMG locus tag ANME2a_02262 or gene ID 2566125492, shown in orange) and DsrA of *Archaeoglobus fulgidus* (PDB 3mm5A, in gray, RMSD=0.93). (**b**) Close-up active site showing differences between DsrA of *A. fulgidus* and Group II Fsr of ANME-2a, in particular Arg98 and Arg170 of DsrA were not conserved in Group II Fsr. The four key residues in substrate positioning are shown by stick representations, and the dashed yellow lines and numbers indicate their distances to sulfite in

Angstrom (shown in yellow). Other co-factors siroheme (white/red/blue) and iron sulfur clusters (yellow/orange) involved in the catalysis are also shown.

(a)

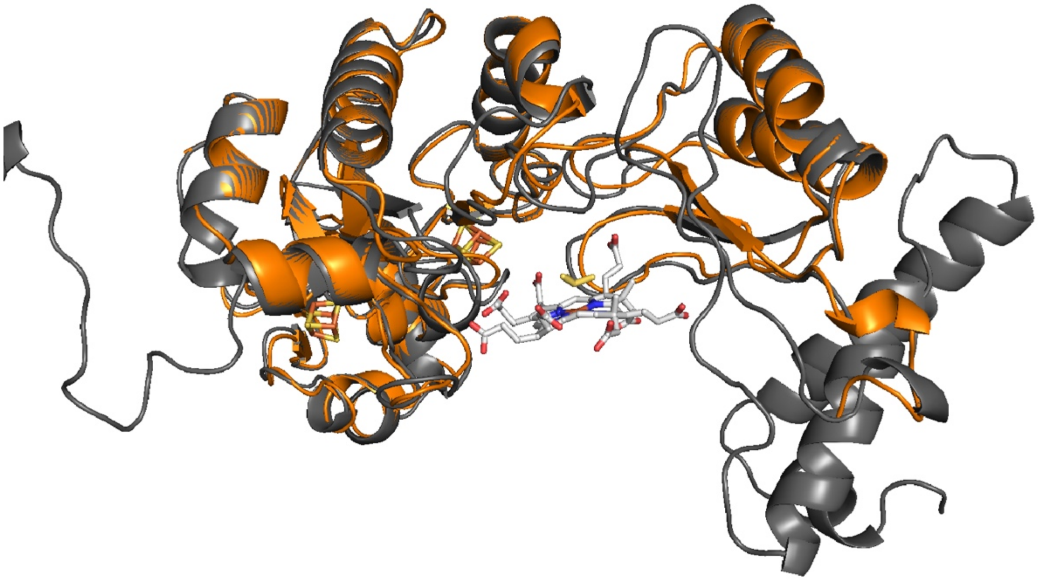

(b)

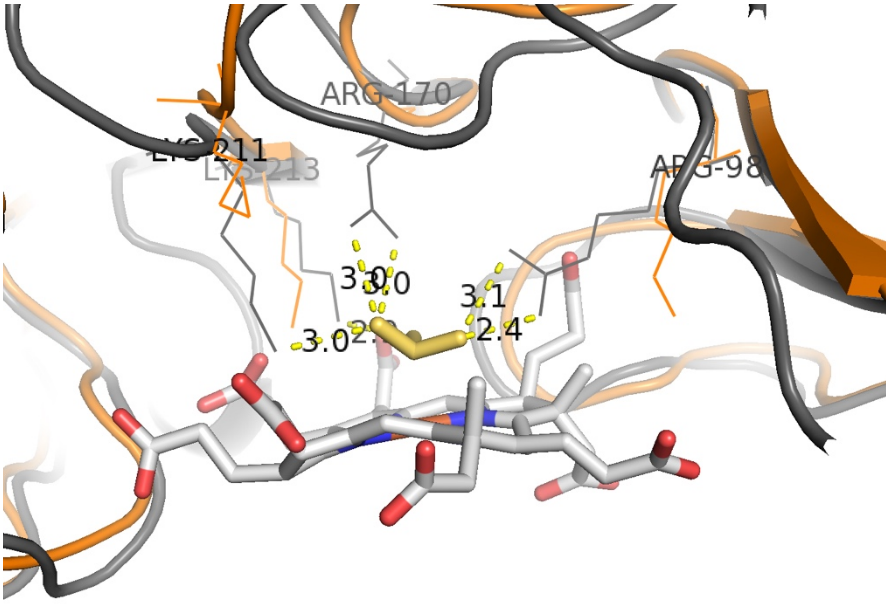

Supplementary Figure 5. Bayesian phylogeny of 615 amino acid residues of Fsr including the environmental clones. Three sets of primers (*=Fsr_GZ_Full_F+Fsr_GZ_full_R, **=Fsr_ANME_259F11 & Fsr_1923R2, ***=Fsr_ANME_265F13 & Fsr_1923R3) were used to amplify Group II Fsr coding regions from 4 methane seep sediment IDs 3730, 5207, 5059 and 5547 as indicated in the clone names. Protein accession numbers from the NCBI database or gene IDs from the IMG database are shown in parentheses. Black dots on the branches represent Bayesian posterior probability values greater than 90%, and scale bar indicates the number of amino acid substitutions per site.

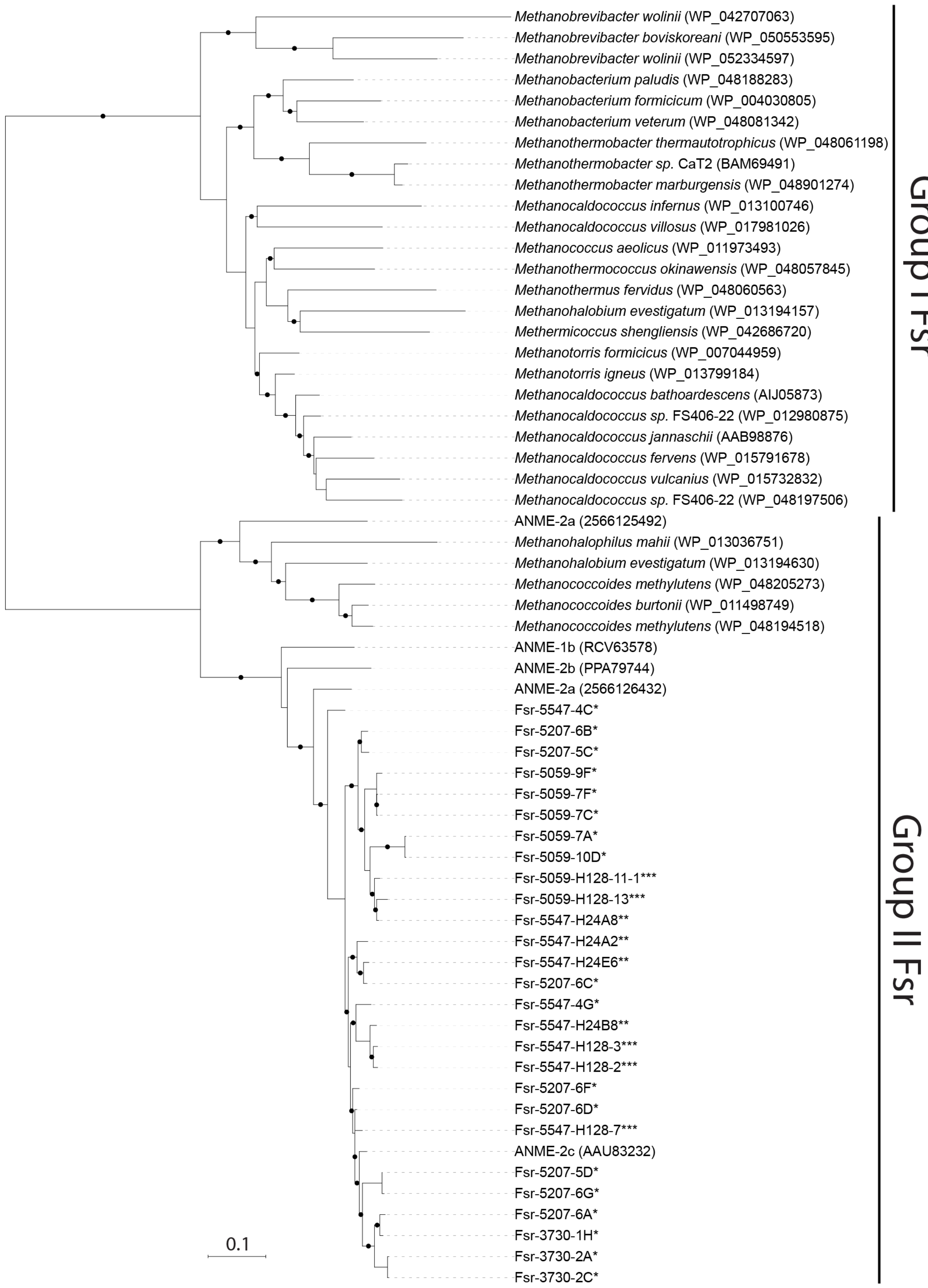

Methanobrevibacter wolinii (WP_042707063)
Methanobrevibacter boviskoreani (WP_050553595)
Methanobrevibacter wolinii (WP_052334597)
Methanobacterium paludis (WP_048188283)
Methanobacterium formicicum (WP_004030805)
Methanobacterium veterum (WP_048081342)
Methanothermobacter thermautotrophicus (WP_048061198)
Methanothermobacter sp. CaT2 (BAM69491)
Methanothermobacter marburgensis (WP_048901274)
Methanocaldococcus infernus (WP_013100746)
Methanocaldococcus villosus (WP_017981026)
Methanococcus aeolicus (WP_011973493)
Methanothermococcus okinawensis (WP_048057845)
Methanothermus fervidus (WP_048060563)
Methanohalobium evestigatum (WP_013194157)
Methermicoccus shengliensis (WP_042686720)
Methanotorris formicicus (WP_007044959)
Methanotorris igneus (WP_013799184)
Methanocaldococcus bathoardescens (AIJ05873)
Methanocaldococcus sp. FS406-22 (WP_012980875)
Methanocaldococcus jannaschii (AAB98876)
Methanocaldococcus fervens (WP_015791678)
Methanocaldococcus vulcanius (WP_015732832)
Methanocaldococcus sp. FS406-22 (WP_048197506)
ANME-2a (2566125492)
Methanohalophilus mahii (WP_013036751)
Methanohalobium evestigatum (WP_013194630)
Methanococcoides methylutens (WP_048205273)
Methanococcoides burtonii (WP_011498749)
Methanococcoides methylutens (WP_048194518)
ANME-1b (RCV63578)
ANME-2b (PPA79744)
ANME-2a (2566126432)
Fsr-5547-4C*
Fsr-5207-6B*
Fsr-5207-5C*
Fsr-5059-9F*
Fsr-5059-7F*
Fsr-5059-7C*
Fsr-5059-7A*
Fsr-5059-10D*
Fsr-5059-H128-11-1***
Fsr-5059-H128-13***
Fsr-5547-H24A8**
Fsr-5547-H24A2**
Fsr-5547-H24E6**
Fsr-5207-6C*
Fsr-5547-4G*
Fsr-5547-H24B8**
Fsr-5547-H128-3***
Fsr-5547-H128-2***
Fsr-5207-6F*
Fsr-5207-6D*
Fsr-5547-H128-7***
ANME-2c (AAU83232)
Fsr-5207-5D*
Fsr-5207-6G*
Fsr-5207-6A*
Fsr-3730-1H*
Fsr-3730-2A*
Fsr-3730-2C*
Group I Fsr
Group II Fsr
0.1

Supplementary Figure 6. Community analysis based on 16s rDNA iTag sequencing of methane seep microcosms amended with different sulfur species. Original = original sediment prior to microcosm experiments, CH4=control microcosm with methane headspace and natural seawater (contains sulfate), N2=control microcosm with nitrogen headspace and natural seawater, Polythionate=CH4 control amended with polythionate, Polysulfide=CH4 control amended with sulfur powder that would form polysulfide upon reaction with sulfide in the microcosm, Sulfite=CH4 control amended with 10 mM sulfite, Thiosulfate=CH4 control amended with 10 mM thiosulfate. The seawater in the microcosms were replaced monthly and new amendments were added. After 6 months, sediments from microcosms were sampled and extracted for 16s rDNA iTag sequencing. No increase in relative abundance of Desulfobacteraceae, which include the dominant SRB partner SEEP-SRB1 group in the sample, was observed in the sulfur amended microcosms.

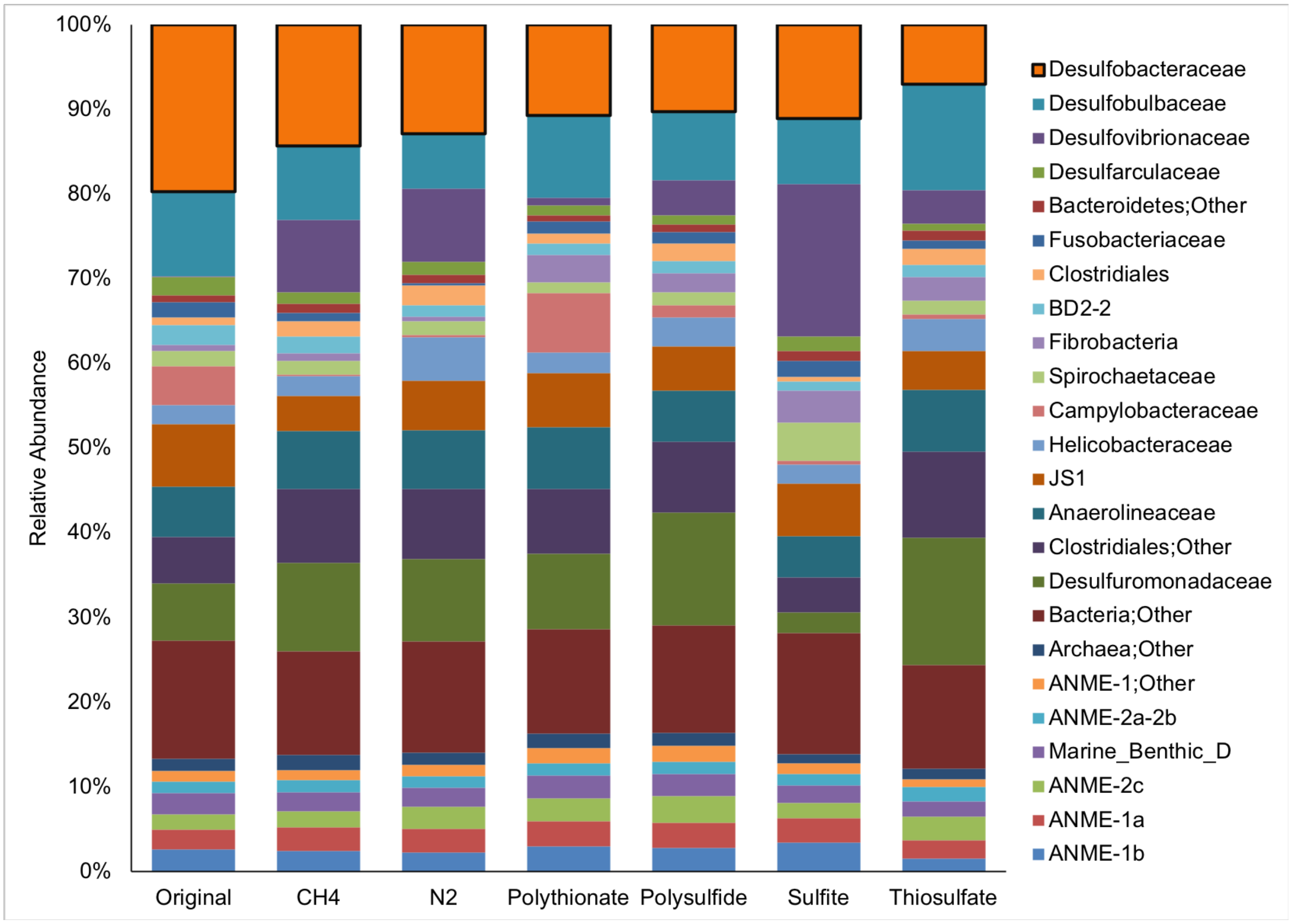

2.2 Supplementary Tables

Supplementary Table 1. Genes for assimilatory or dissimilatory sulfate reduction in the genomes of ANMEs and representative methanogens. (Attached Separately)

Supplementary Table 2. Proteins highly expressed in the bulk methane seep metaproteome. Proteins associated with sulfur pathways are highlighted in bold. Protein expression levels are reported as averaged normalized spectral counts (nSpc) in environmental metaproteomes. The searches used a

database containing multiple methane seep metagenomes and genomes of cultured representatives. (Attached Separately)

Supplementary Table 3. Sample information on the methane seep sediments used in this study.

Sediment ID	Location	Research cruise	Collection Date	Water depth (m)	Site description
**5133**	44°40.02'N, 125°6.00'W	AT 18-10, R/V Atlantis, ROV JASON II	September 2011	597	Hydrate Ridge, Oregon USA, below a white microbial mat
**7142**	33°47.33'N, 118°40.09'W	R/V Western Flyer, ROV Doc Ricketts	May 2013	860.5	Santa Monica Basin, California USA, below a white microbial mat. The dominant ANME lineage in the same is ANME-2a and ANME-2c, as described in Scheller *et al.* 2016.
**3730**	44°43.09'N, 125°9.14'W	AT 15-38, R/V Atlantis, HOV Alvin	August 2010	776	Hydrate Ridge, Oregon USA, below a Calyptogena clam bed
**5059**	44°40.19'N, 125°5.88'W	AT 18-10, R/V Atlantis, ROV JASON II	September 2011	595	Hydrate Ridge, Oregon USA, below a clam field
**5207**	44°40.02'N, 125°6.00'W	AT 18-10, R/V Atlantis, ROV JASON II	September 2011	601	Hydrate Ridge, Oregon USA, below a white microbial mat
**5547**	44°34.19'N, 125°8.86'W	AT 18-10, R/V Atlantis, ROV JASON II	September 2011	775	Hydrate Ridge, Oregon USA, below a white microbial mat

Supplementary Table 4. PCR primers used to amplify Group II *fsr* of ANME from methane seep sediment samples.

Primer name	Primer sequence	Note
Fsr_ANME_259F11	TGTACMYTCTGCGGCGC	Degenerate primer
Fsr_ANME_265F13	YTMTGCGGYGCATGTGC	Degenerate primer
Fsr_1923R2	CKGAYRCACCATCCACA	Degenerate primer
Fsr_1923R3	CTGATRCACCAKCCACA	Degenerate primer
Fsr_GZ_full_F	GCGCATGCATATGGCAAA CGAAGAATATAAATGG	Contains NsiI digest site ATGCAT
Fsr_GZ_full_R	TAATGGATCCTCACACCT GATCCAGAACCTCT	Contains the reverse complement of BamHI digest site GGATCC

Supplementary Table 5. Specific search for sulfur pathway proteins of *Ca*. Methanoperedens in methane seep metaproteomes. The searches used a streamlined database containing only proteins of interest, and peptide fragmentation spectra of proteins found to be expressed were also manually validated in Supplementary Data Sheet 1.

			**Averaged Normalized Spectral Counts (nSpc) in Environmental Metaproteomes**				
Protein accession	**Description**	**Organism**	**Hydrate Ridge**	**Santa Monica_0-4cm**	**Santa Monica_8-12cm**	**Eel River_0-10cm**	**Eel River_10-20cm**
WP_052368917	CysN	Ca. Methanoperedens nitroreducens	2648.5	b.d.	b.d.	b.d.	b.d.
KCZ70985	aSir	Ca. Methanoperedens nitroreducens	b.d.	100.3	b.d.	b.d.	b.d.
WP_048089148	Putative PAPS reductase	Ca. Methanoperedens nitroreducens	829.8	b.d.	b.d.	b.d.	b.d.
WP_048090348	APS reductase	Ca. Methanoperedens nitroreducens	b.d.	b.d.	b.d.	46.2	b.d.
KPQ43960	Putative APS reductase	Ca. Methanoperedens sp. BLZ1	b.d.	b.d.	b.d.	38.4	b.d.
KPQ44278	Group III Dsr-LP	Ca. Methanoperedens sp. BLZ1	b.d.	b.d.	b.d.	67.9	b.d.
**b.d. = below detection limits of mass spectrometry**							

2.3 Supplementary Data Sheet

Supplementary Data Sheet 1. Mass Spectra of ANME proteins expressed in the methane seep metaproteome. Protein accession numbers from the NCBI database or gene IDs from the IMG database that corresponds to Table 1 and Supplementary Table 5 are indicated on the left. Amongst the different types of cleavages that occur during peptide fragmentation, only peptides determined from b-type and y-type ions are shown in purple and blue, respectively. The peptide sequence from b-type ions was determined by charge remaining on the N-terminal portion of the peptide, whereas the peptide sequence from y-type ions was determined by charge remaining on the C-terminal portion of the peptide, and the sequence is shown in reverse. The determined sequences match in both cases confirming the detection of specific peptides in the metaproteome.

RCV62684

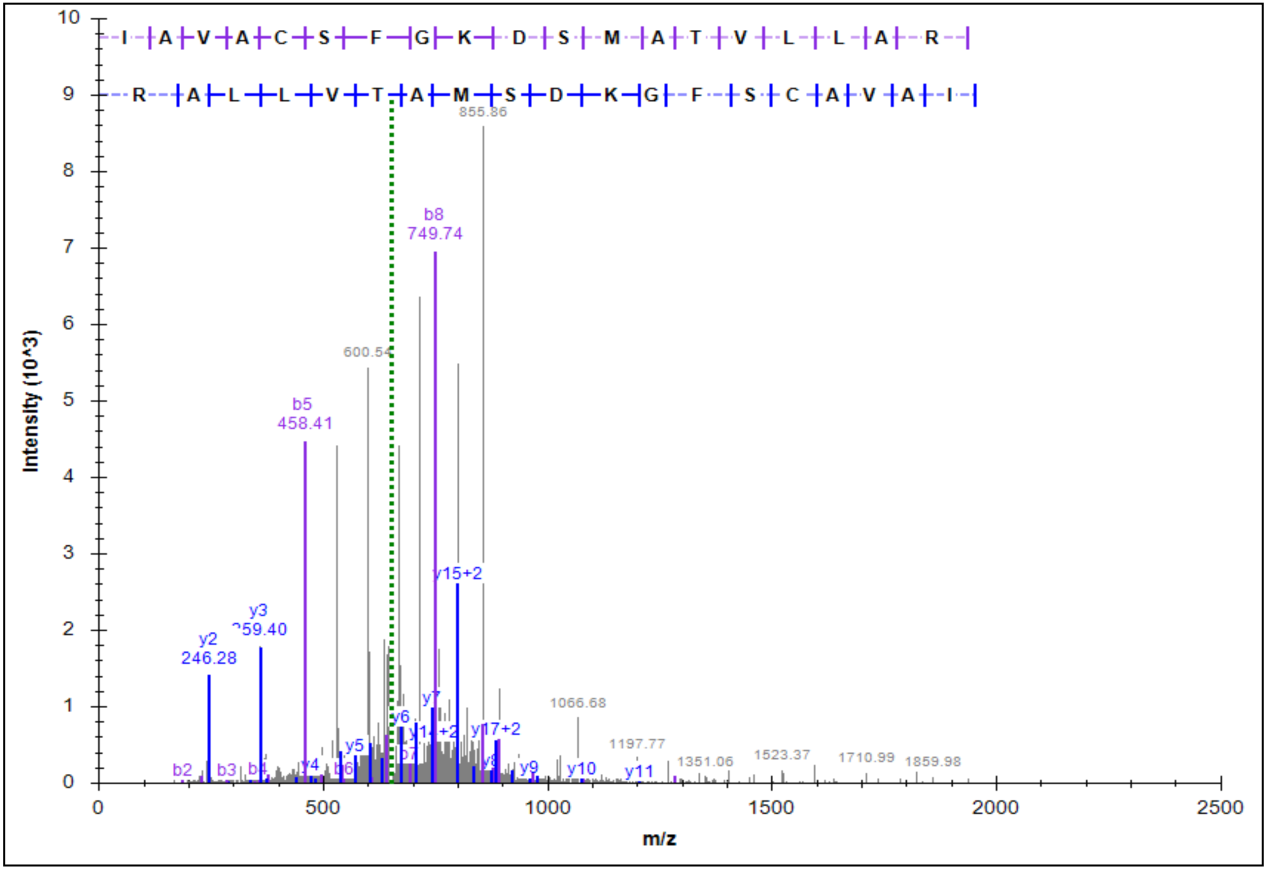
I A V A C S F G K D S M A T V L L A R
R A L L V T A M S D K G F S C A V A I
855.86
b8
749.74
600.54
b5
458.41
y15+2
y3
359.40
y2
246.28
1066.68
y7
y6
y17+2
1197.77
y5
1351.06
1523.37
1710.99
1859.98
b2
b3
b4
y4
b6
y9
y10
y11
Intensity (10^3)
m/z

RCV63267

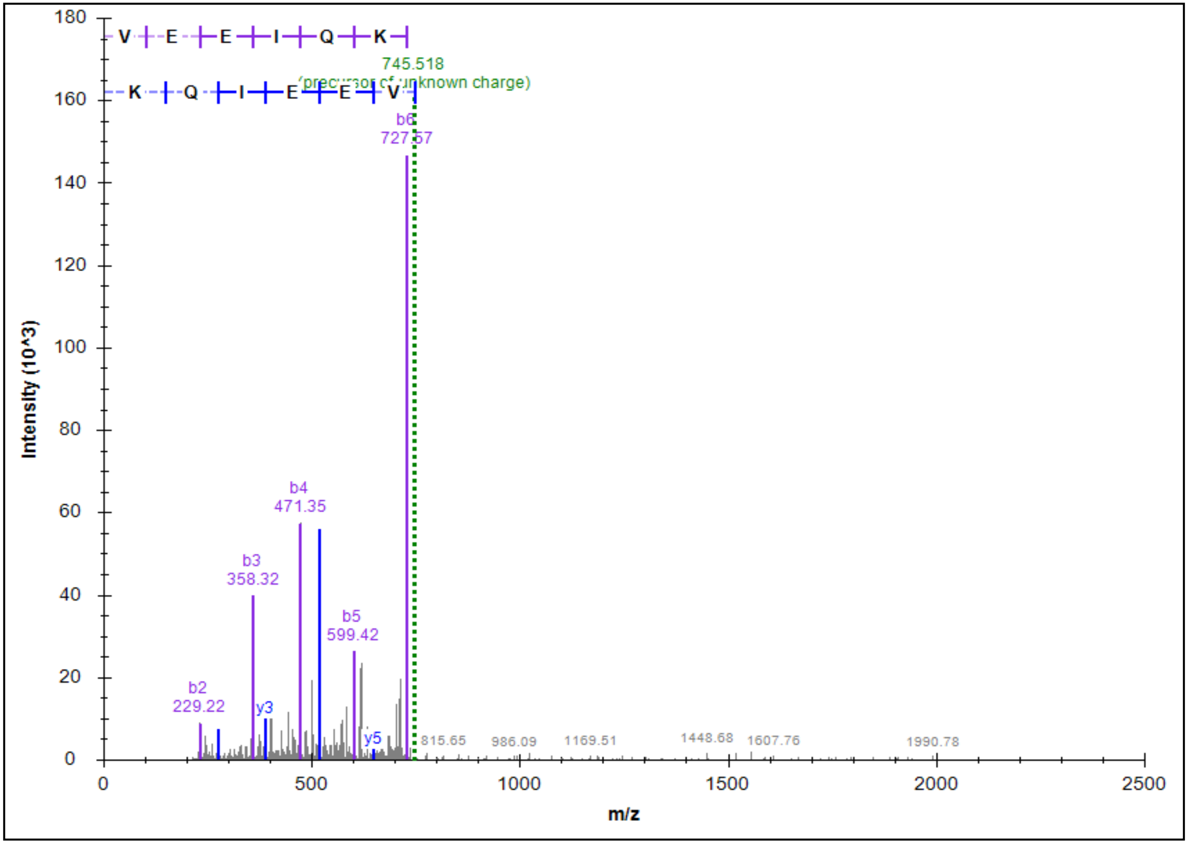

V E E I Q K
K Q I E E V
745.518
(precursor of unknown charge)
b6
727.57
b4
471.35
b3
358.32
b5
599.42
b2
229.22
y3
y5
815.65
986.09
1169.51
1448.68
1607.76
1990.78
Intensity (10^3)
m/z

RCV64987

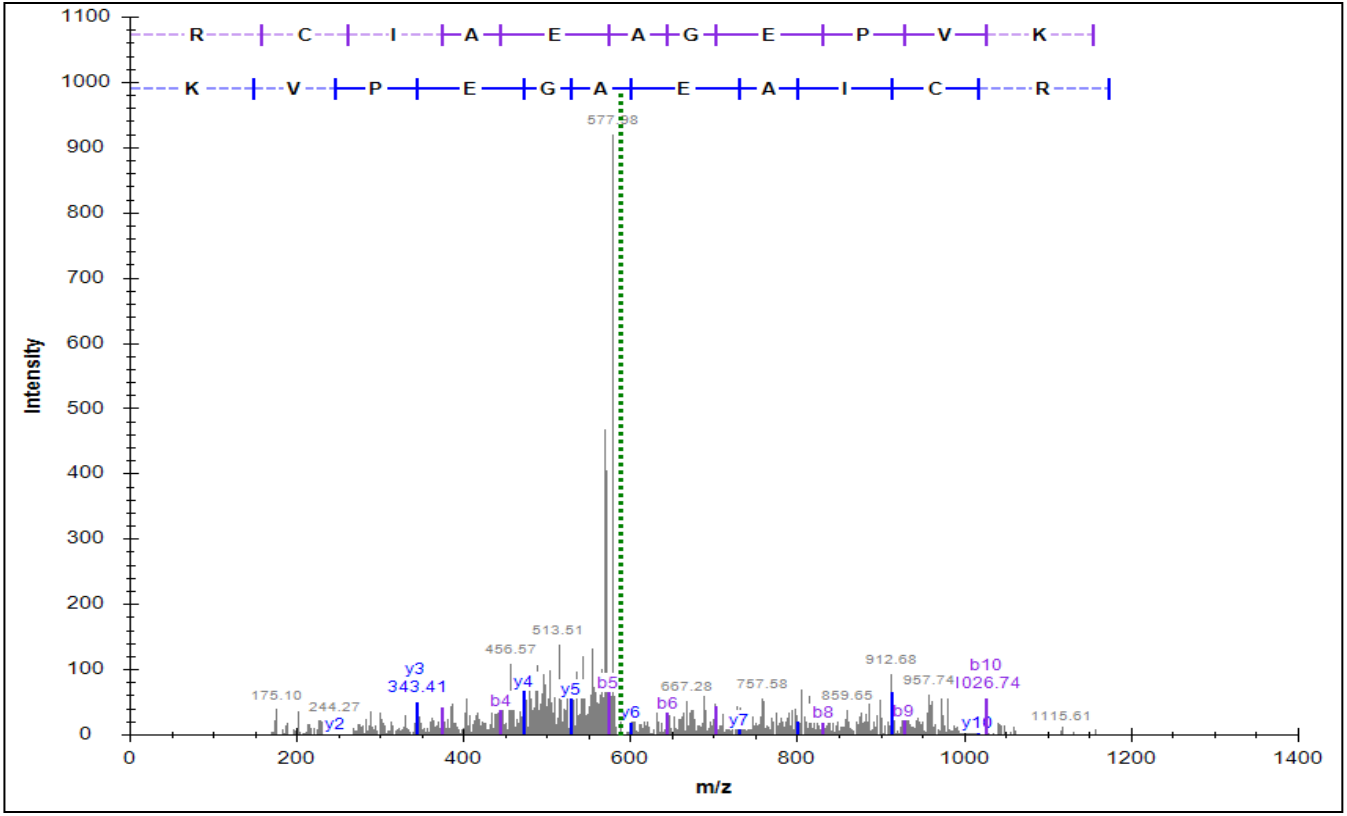

R C I A E A G E P V K
K V P E G A E A I C R
577.98
513.51
456.57
175.10
244.27
y2
y3
343.41
y4
b4
y5
b5
y6
b6
667.28
757.58
y7
859.65
b8
912.68
957.74
b9
b10
1026.74
y10
1115.61
Intensity
m/z

CBH38748

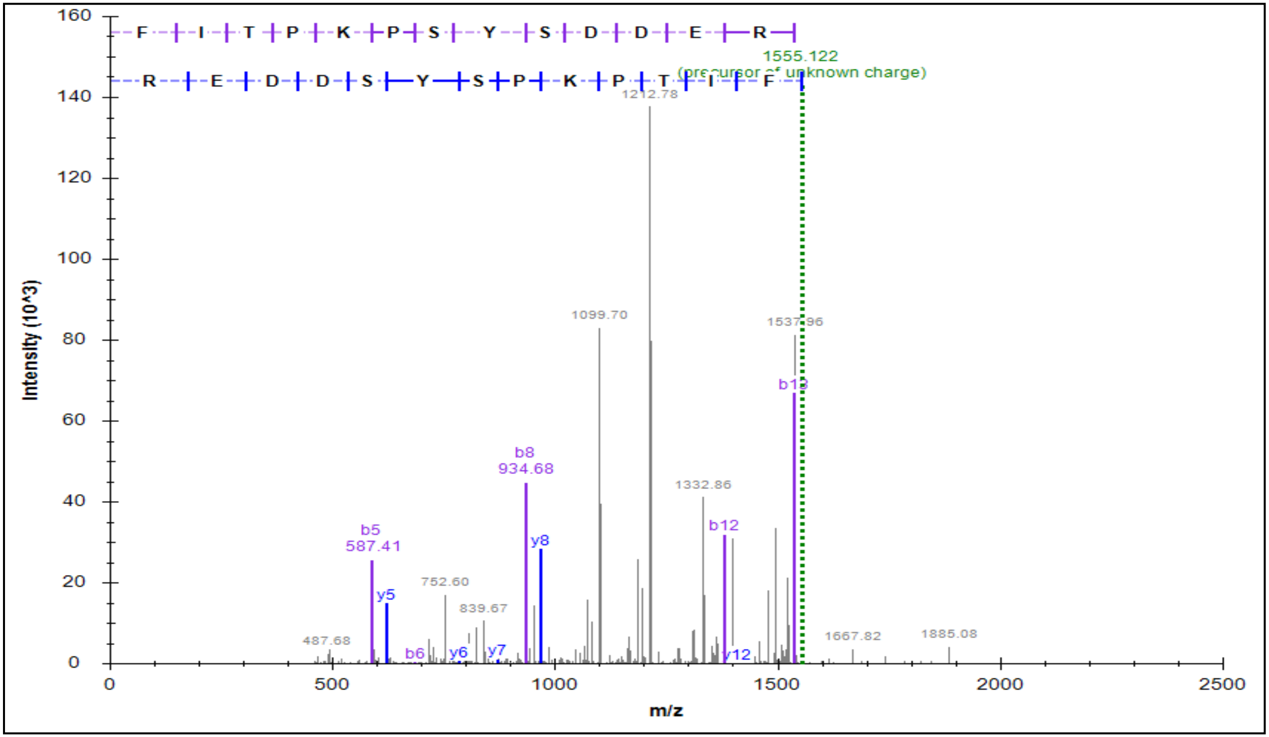

F I T P K P S Y S D D E R
R E D D S Y S P K P T I F
1555.122
(precursor of unknown charge)
1212.78
1099.70
1537.96
b13
b8
934.68
1332.86
b12
b5
587.41
y8
y5
752.60
839.67
487.68
b6
y6
y7
y12
1667.82
1885.08
Intensity (10^3)
m/z
0
20
40
60
80
100
120
140
160
0
500
1000
1500
2000
2500

2566123967

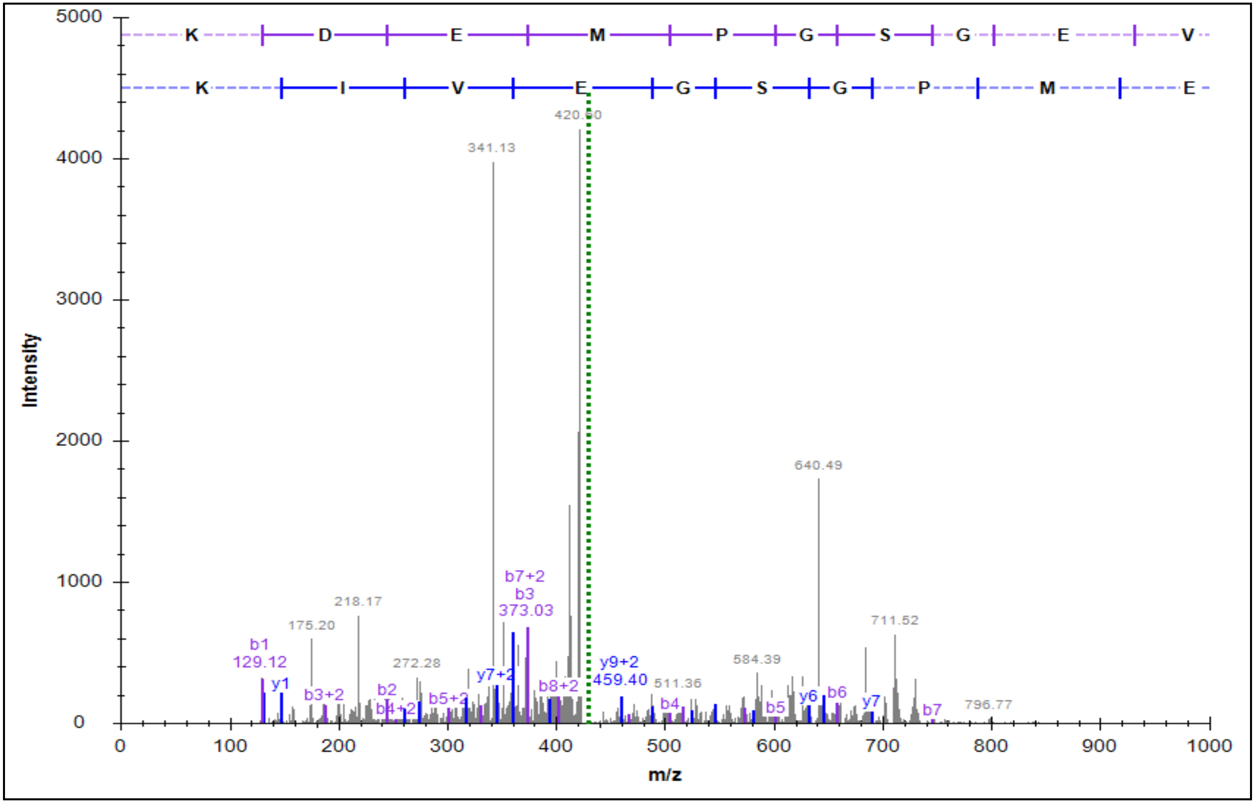
K D E M P G S G E V
K I V E G S G P M E
Intensity
m/z
5000
4000
3000
2000
1000
0
0
100
200
300
400
500
600
700
800
900
1000
420.60
341.13
640.49
711.52
218.17
175.20
272.28
584.39
511.36
796.77
b1
129.12
y1
b3+2
b2
b4+2
b5+2
y7+2
b7+2
b3
373.03
b8+2
y9+2
459.40
b4
b5
y6
b6
y7
b7

PPA79744

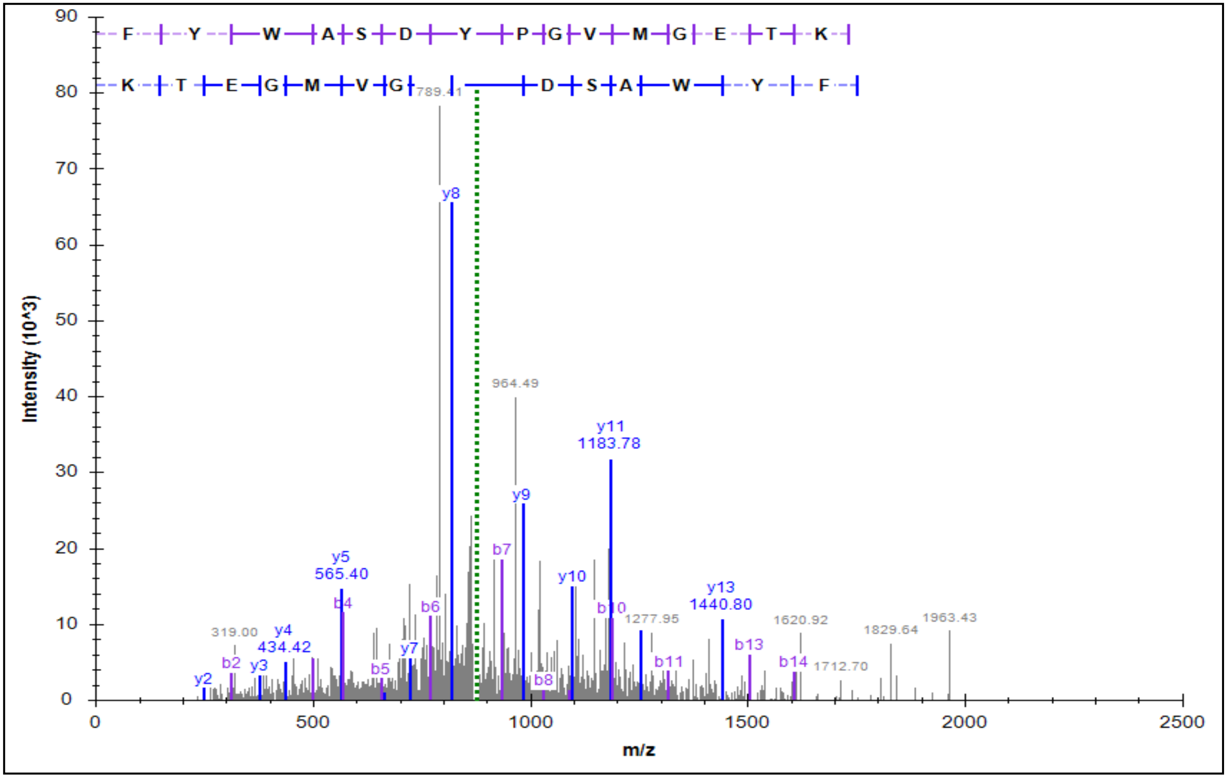
F Y W A S D Y P G V M G E T K
K T E G M V G D S A W Y F
Intensity (10^3)
m/z
789.41
964.49
y8
y11
1183.78
y9
b7
y5
565.40
b4
b6
y10
b10
y13
1440.80
1277.95
1620.92
1963.43
1829.64
319.00
y4
434.42
y7
b2
y2
y3
b5
b8
b11
b13
b14
1712.70

PPA80122

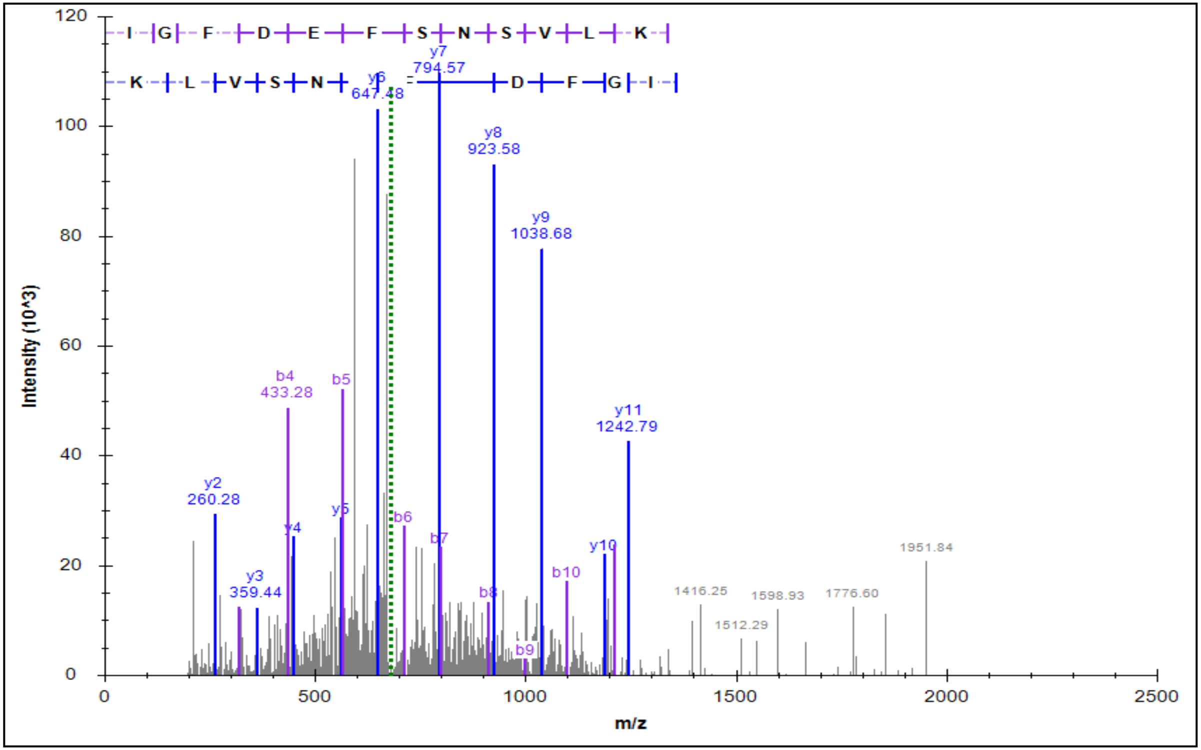

I G F D E F S N S V L K
K L V S N D F G I
y2 260.28
y3 359.44
y4
b4 433.28
b5
y5
y6 647.48
y7 794.57
b6
b7
y8 923.58
b8
b9
y9 1038.68
b10
y10
y11 1242.79
1416.25
1512.29
1598.93
1776.60
1951.84
Intensity (10^3)
m/z
0
20
40
60
80
100
120
500
1000
1500
2000
2500

PPA80122

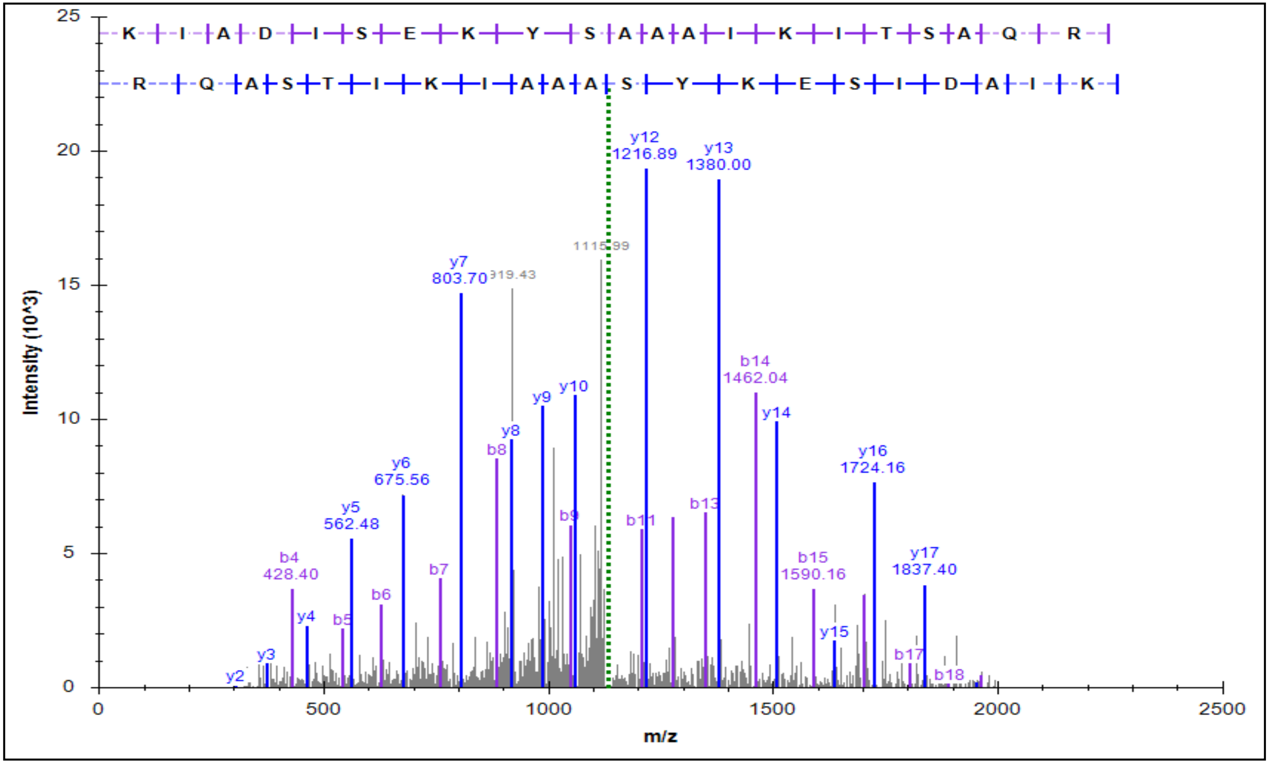
K I A D I S E K Y S A A A I K I T S A Q R
R Q A S T I K I A A A S Y K E S I D A I K
Intensity (10^3)
m/z
y12 1216.89
y13 1380.00
1115.99
y7 803.70
919.43
b14 1462.04
y14
y16 1724.16
y17 1837.40
b15 1590.16
y15
b17
b18
y9
y10
y8
b8
b9
b11
b13
y6 675.56
y5 562.48
b4 428.40
y4
b5
b6
b7
y3
y2

AAU83232

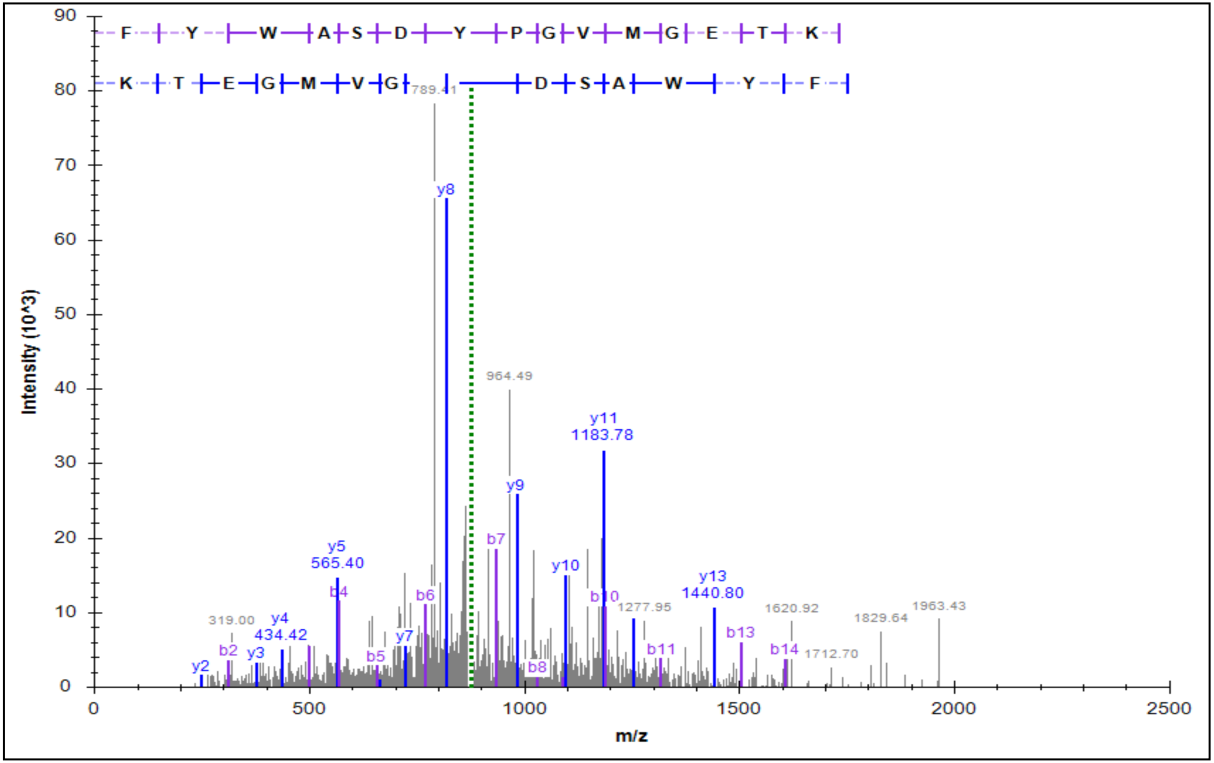
F Y W A S D Y P G V M G E T K
K T E G M V G 789.41 D S A W Y F
Intensity (10^3)
m/z
y8
964.49
y11
1183.78
y9
b7
y5
565.40
y10
b4
b6
b10
1277.95
y13
1440.80
1620.92
1963.43
1829.64
319.00
y4
434.42
y7
b13
b2
y3
b5
b11
b14
1712.70
y2
b8

AAU83232

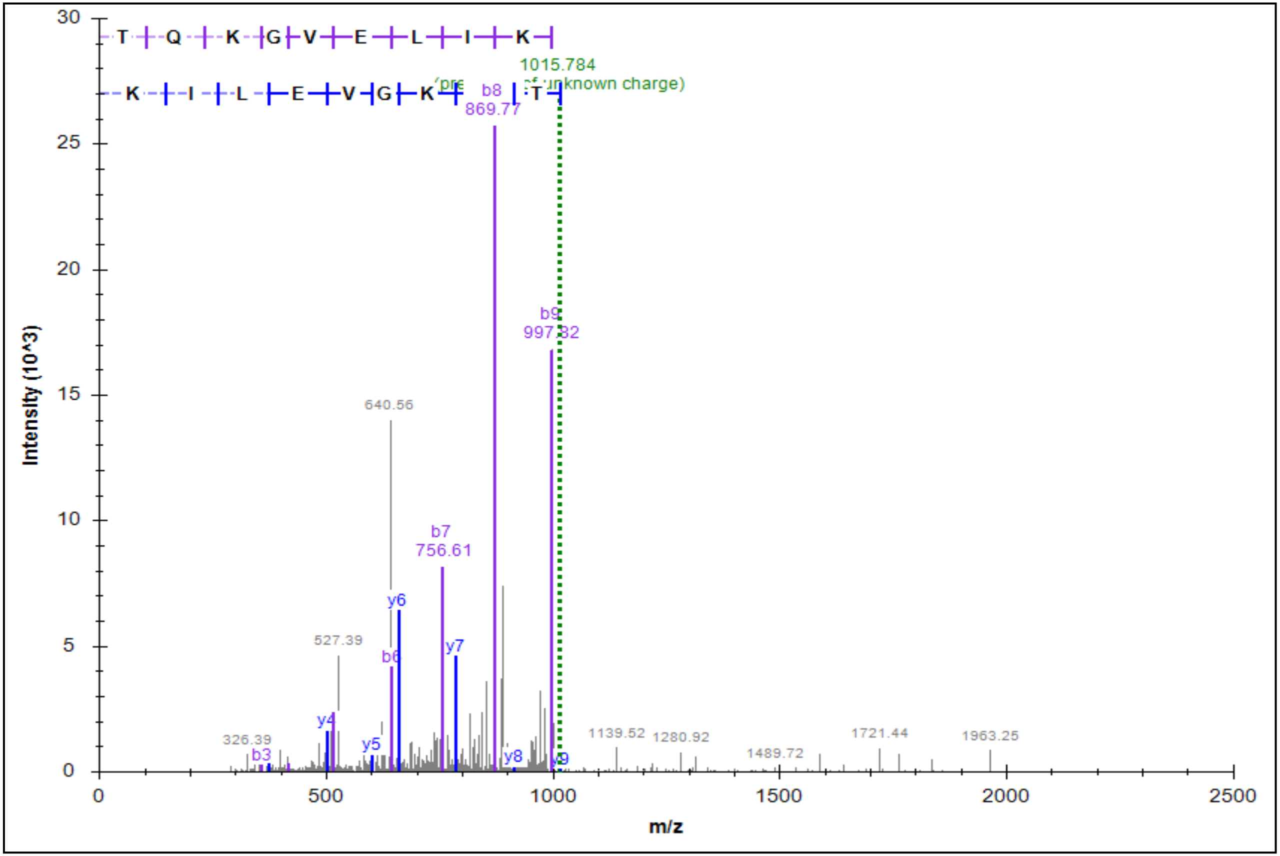

T Q K G V E L I K
K I L E V G K T
1015.784
(precursor of unknown charge)
b8
869.77
b9
997.32
640.56
b7
756.61
y6
527.39
b6
y7
y4
326.39
y5
b3
y8
y9
1139.52
1280.92
1489.72
1721.44
1963.25
Intensity (10^3)
m/z
0
5
10
15
20
25
30
500
1000
1500
2000
2500

AAU83223

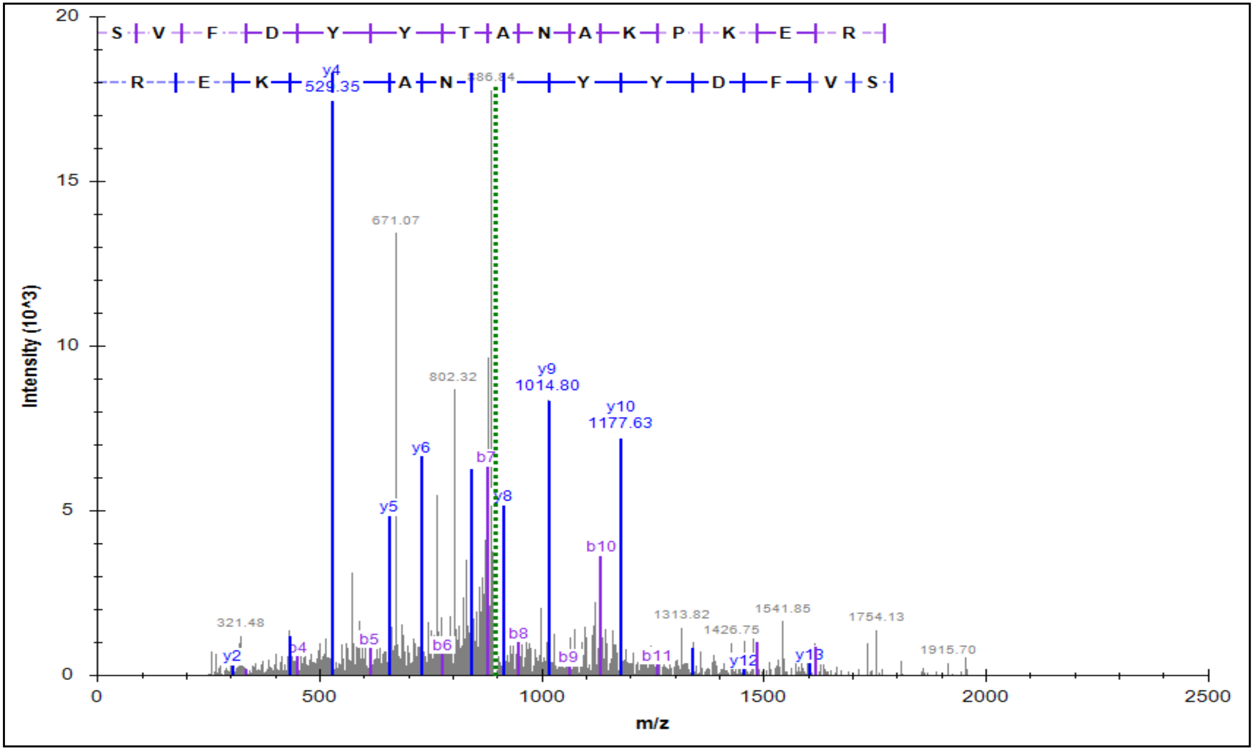
S V F D Y Y T A N A K P K E R
R E K A N Y Y D F V S
y4
529.35
886.84
671.07
802.32
y9
1014.80
y10
1177.63
y6
y5
b7
y8
b10
321.48
1313.82
1426.75
1541.85
1754.13
1915.70
y2
b4
b5
b6
b8
b9
b11
y12
y13
Intensity (10^3)
m/z
0
5
10
15
20
500
1000
1500
2000
2500

AAU83223

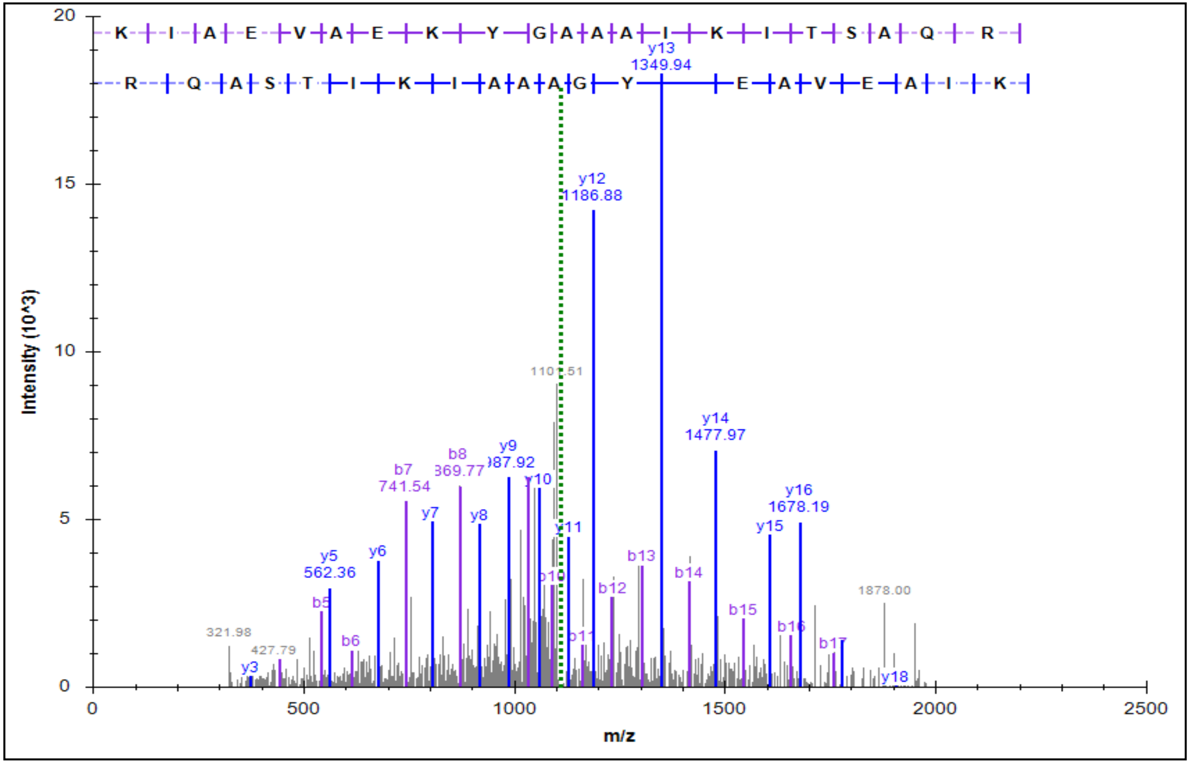

K I A E V A E K Y G A A A I K I T S A Q R
R Q A S T I K I A A A G Y E A V E A I K
y13
1349.94
y12
1186.88
y14
1477.97
y16
1678.19
y15
y11
y10
y9
b8
b7
741.54
y7
y8
y6
y5
562.36
b5
b6
b13
b12
b14
b15
b16
b17
b11
y18
y3
321.98
427.79
1878.00
Intensity (10^3)
m/z
0
500
1000
1500
2000
2500
5
10
15
20

WP_052368917

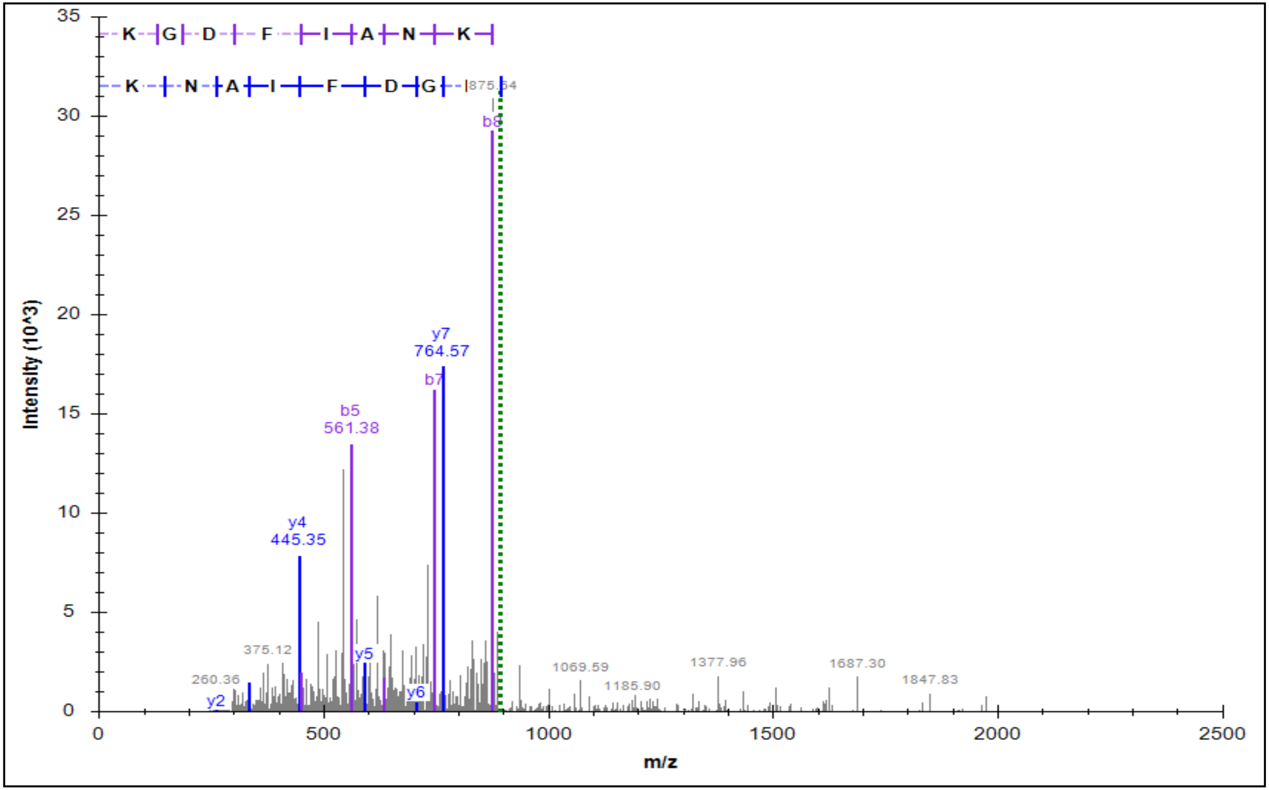

KCZ70985

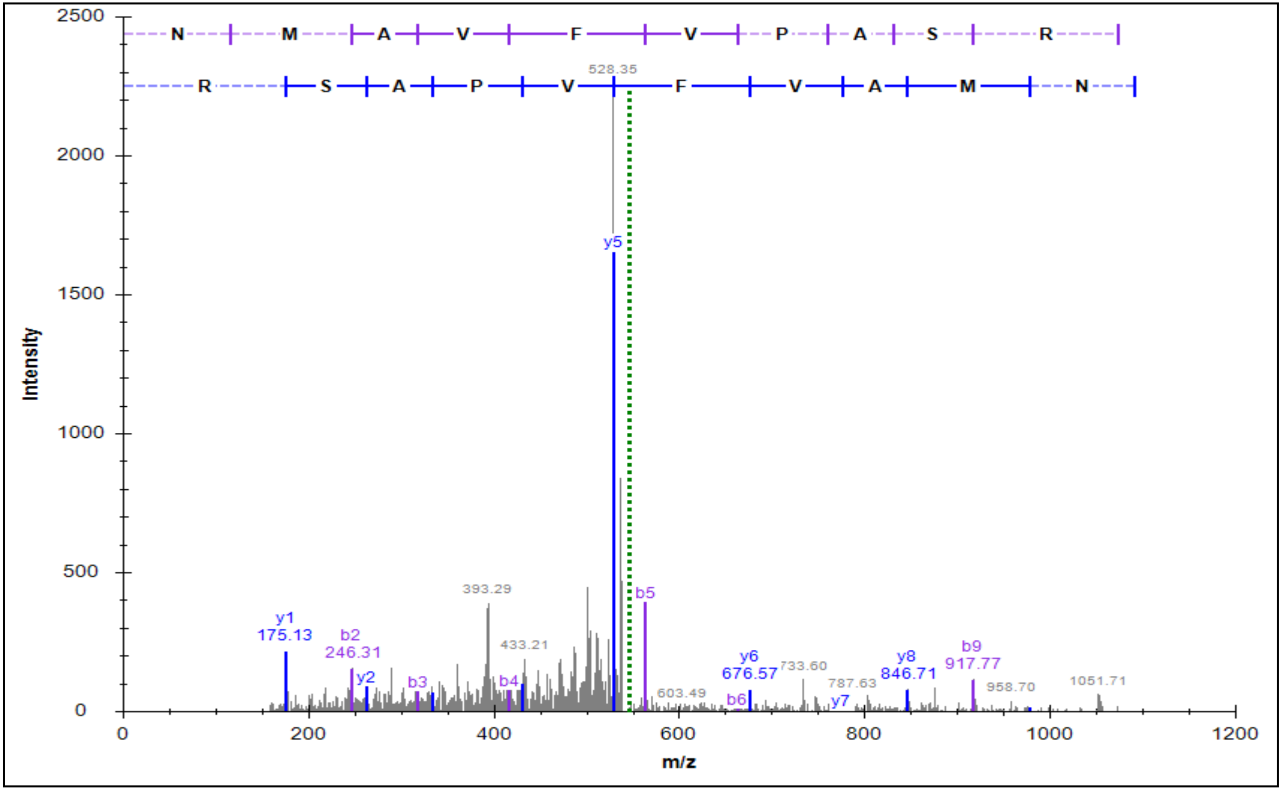

N M A V F V P A S R
R S A P V F V A M N
528.35
y5
b5
393.29
433.21
y1
175.13
b2
246.31
y2
b3
b4
603.49
y6
676.57
733.60
b6
787.63
y7
y8
846.71
b9
917.77
958.70
1051.71
2500
2000
1500
1000
500
0
Intensity
0
200
400
600
800
1000
1200
m/z

2515321511

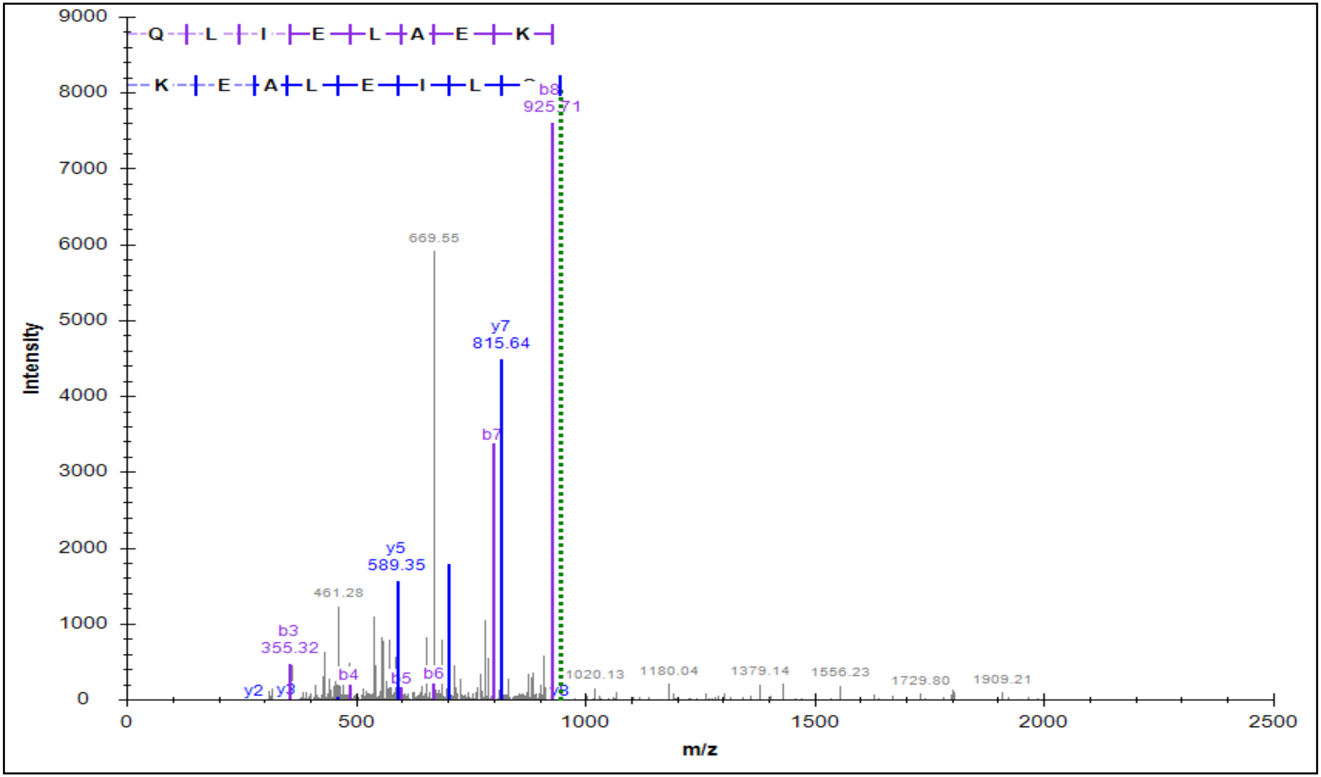

Q L I E L A E K
K E A L E I L
Intensity
m/z
669.55
y7
815.64
b7
b8
925.71
y5
589.35
461.28
b3
355.32
b4
b5
b6
y2
y3
y8
1020.13
1180.04
1379.14
1556.23
1729.80
1909.21

2515322433

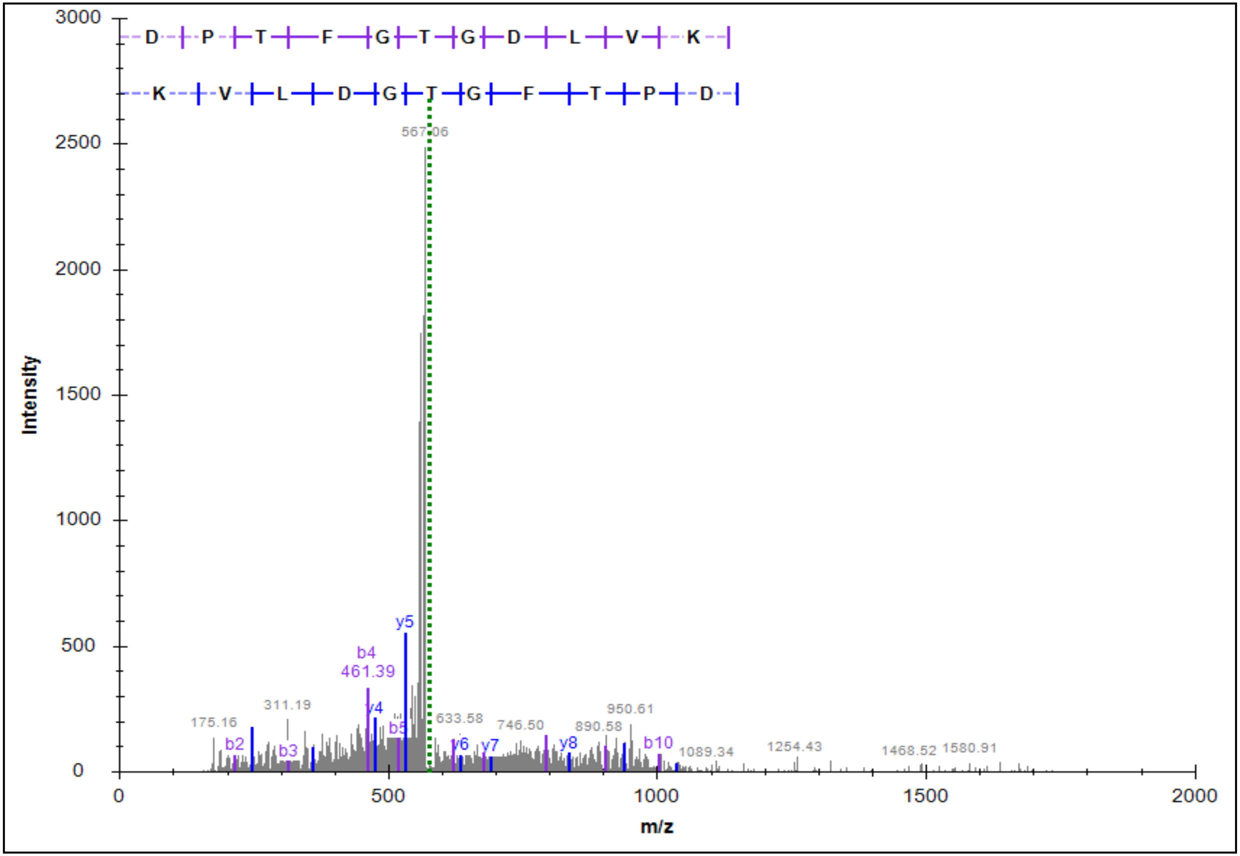

D P T F G T G D L V K
K V L D G T G F T P D
567.06
y5
b4
461.39
y4
b5
311.19
175.16
b2
b3
633.58
y6
y7
746.50
890.58
y8
950.61
b10
1089.34
1254.43
1468.52
1580.91
Intensity
m/z
0
500
1000
1500
2000
2500
3000

KPQ43960

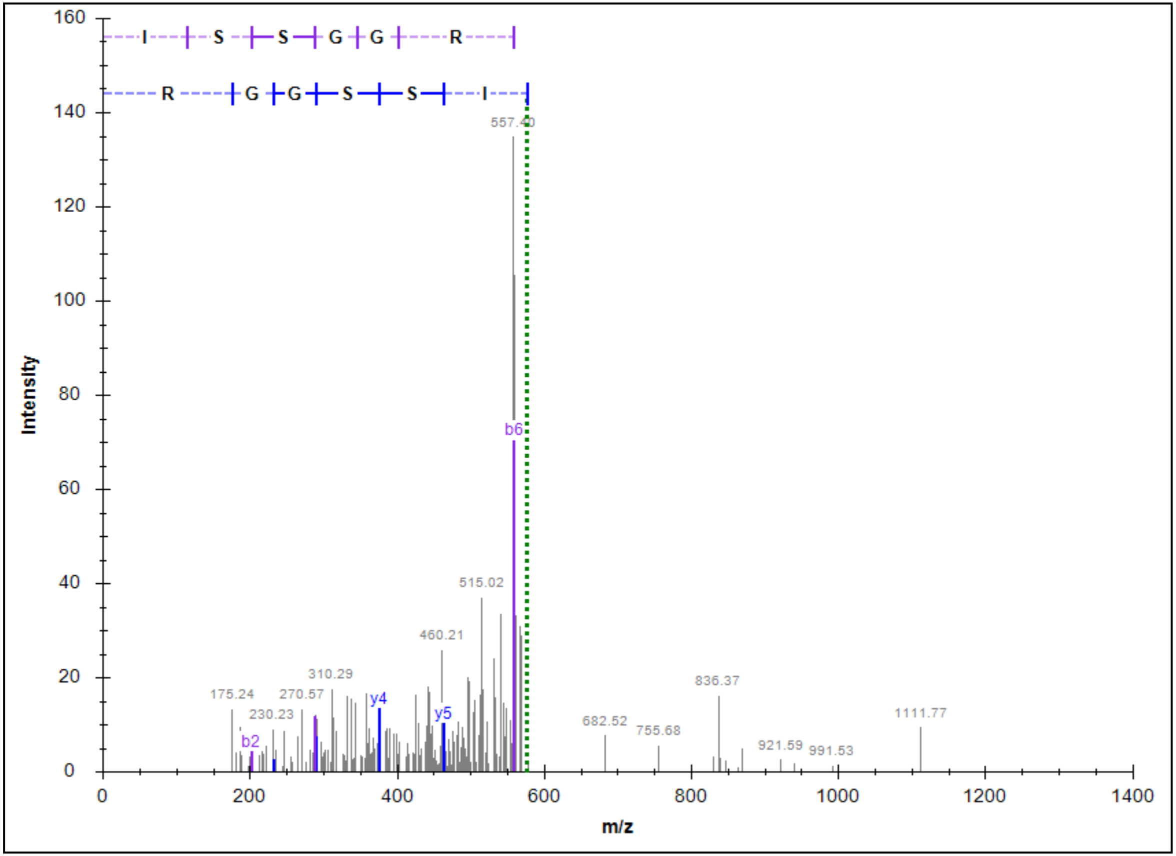
I S S G G R
R G G S S I
557.40
b6
515.02
460.21
310.29
175.24
270.57
230.23
b2
y4
y5
682.52
755.68
836.37
921.59
991.53
1111.77
Intensity
m/z
0
20
40
60
80
100
120
140
160
200
400
600
800
1000
1200
1400

KPQ44278

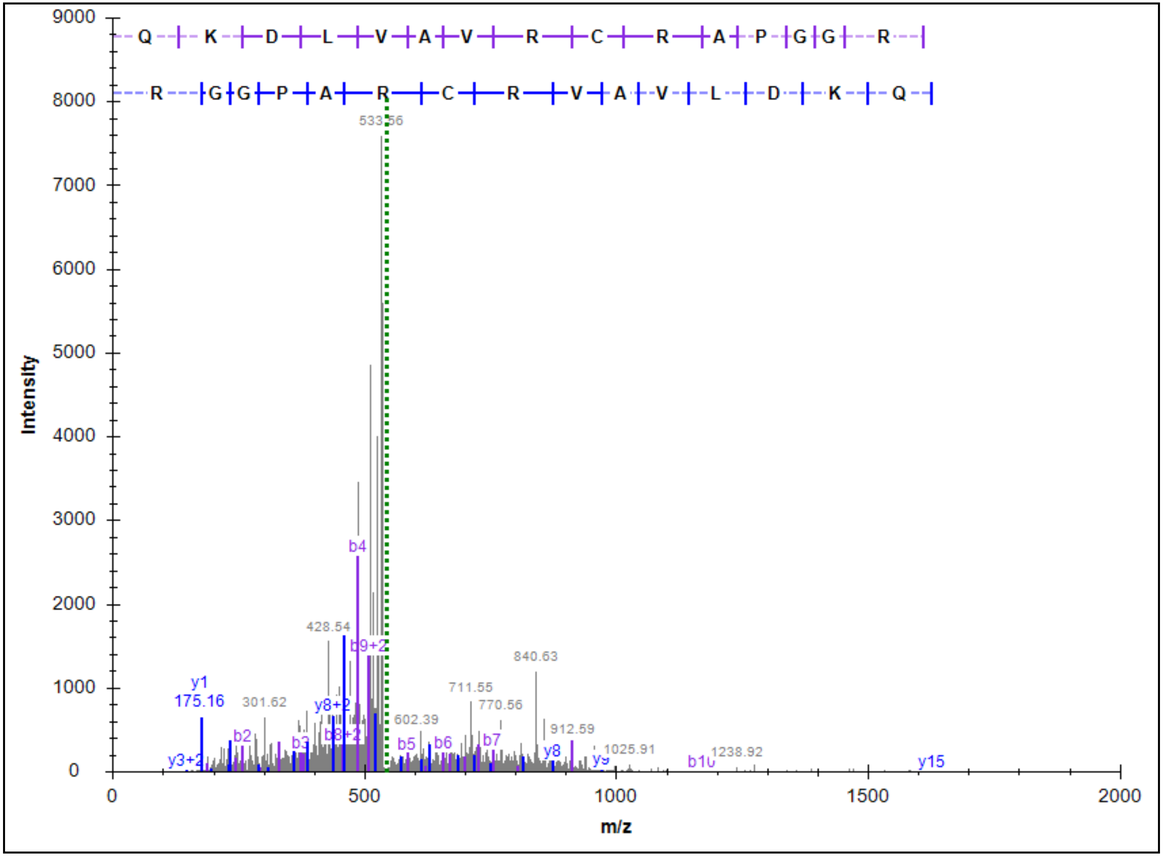

Q K D L V A V R C R A P G G R
R G G P A R C R V A V L D K Q
533.56
b4
428.54
b9+2
840.63
y1
175.16
301.62
y8+2
711.55
770.56
602.39
912.59
b2
b3
b8+2
b5
b6
b7
y8
1025.91
y3+2
y9
b10
1238.92
y15
Intensity
m/z
0
1000
2000
3000
4000
5000
6000
7000
8000
9000
0
500
1000
1500
2000

MH823235

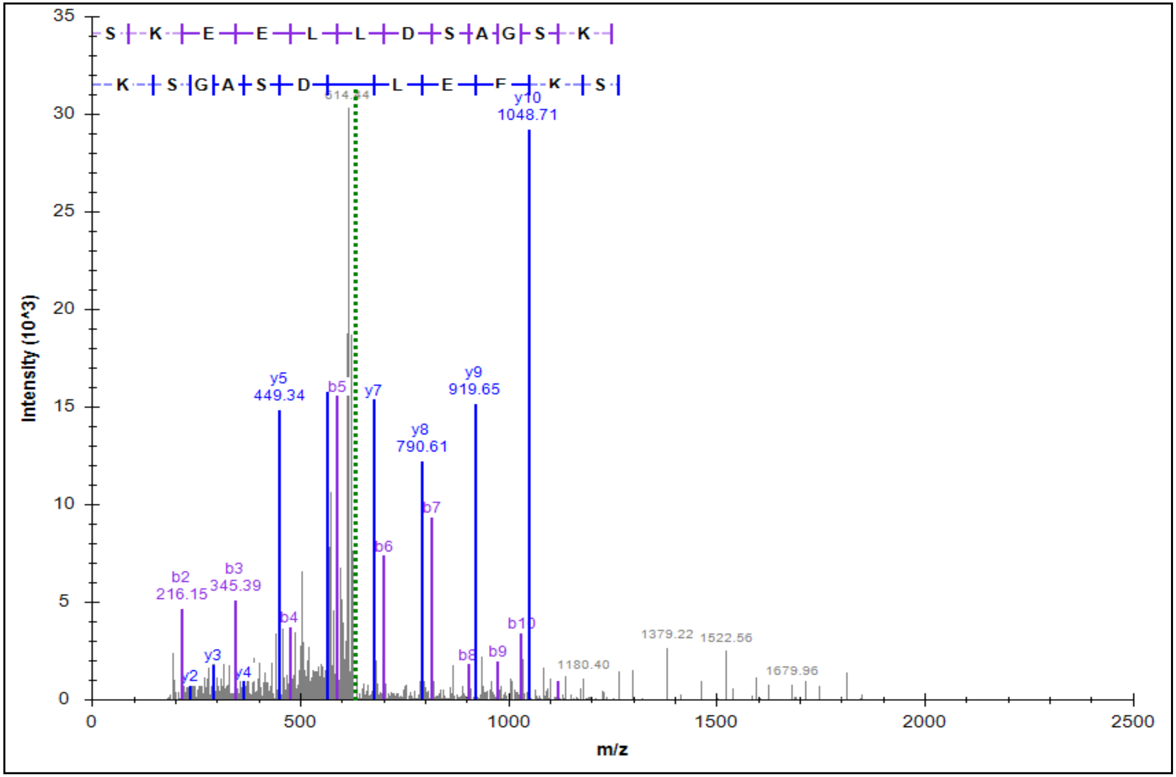

S K E E L L D S A G S K
K S G A S D L E F K S
y10 1048.71
y5 449.34
b5
y7
y9 919.65
y8 790.61
b7
b6
b2 216.15
b3 345.39
b4
y3
y2
y4
b8
b9
b10
1180.40
1379.22
1522.56
1679.96
Intensity (10^3)
m/z

MH823235

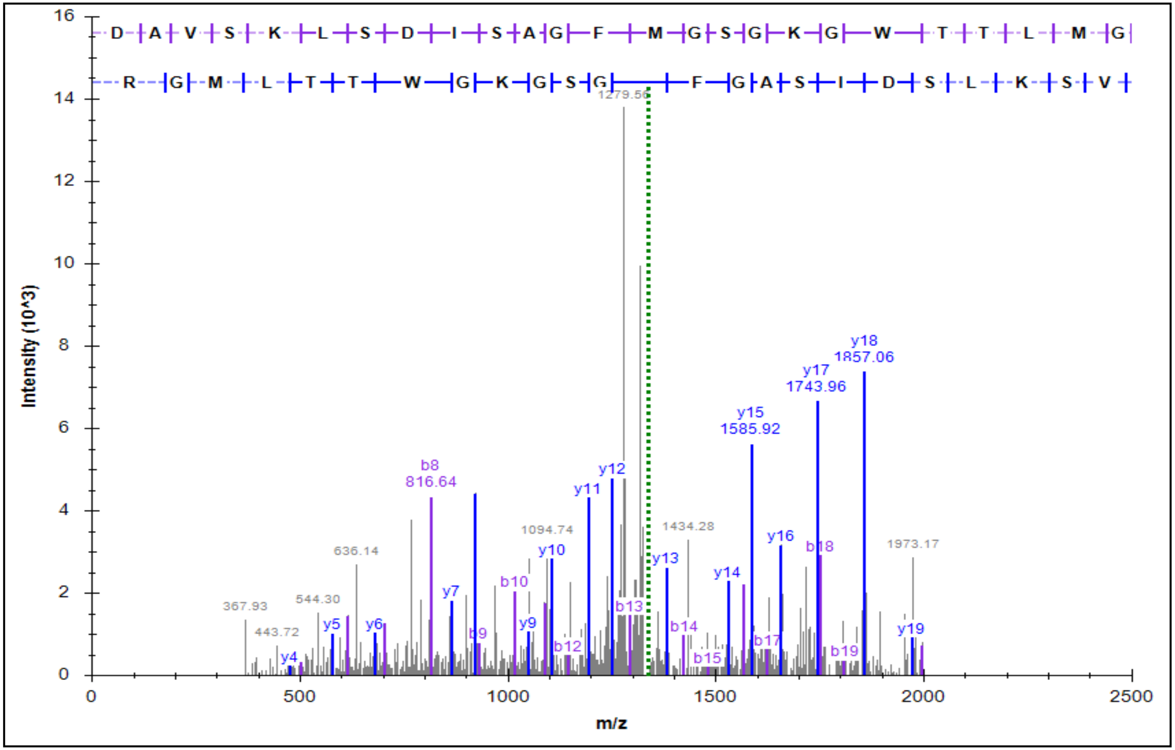
D A V S K L S D I S A G F M G S G K G W T T L M G
R G M L T T W G K G S G F G A S I D S L K S V
1279.56
Intensity (10^3)
m/z
b8 816.64
y18 1857.06
y17 1743.96
y15 1585.92
y11
y12
y10
1094.74
1434.28
1973.17
636.14
544.30
367.93
443.72
y4
y5
y6
y7
b9
b10
y9
b12
b13
y13
b14
b15
y14
y16
b17
b18
b19
y19

MH823238

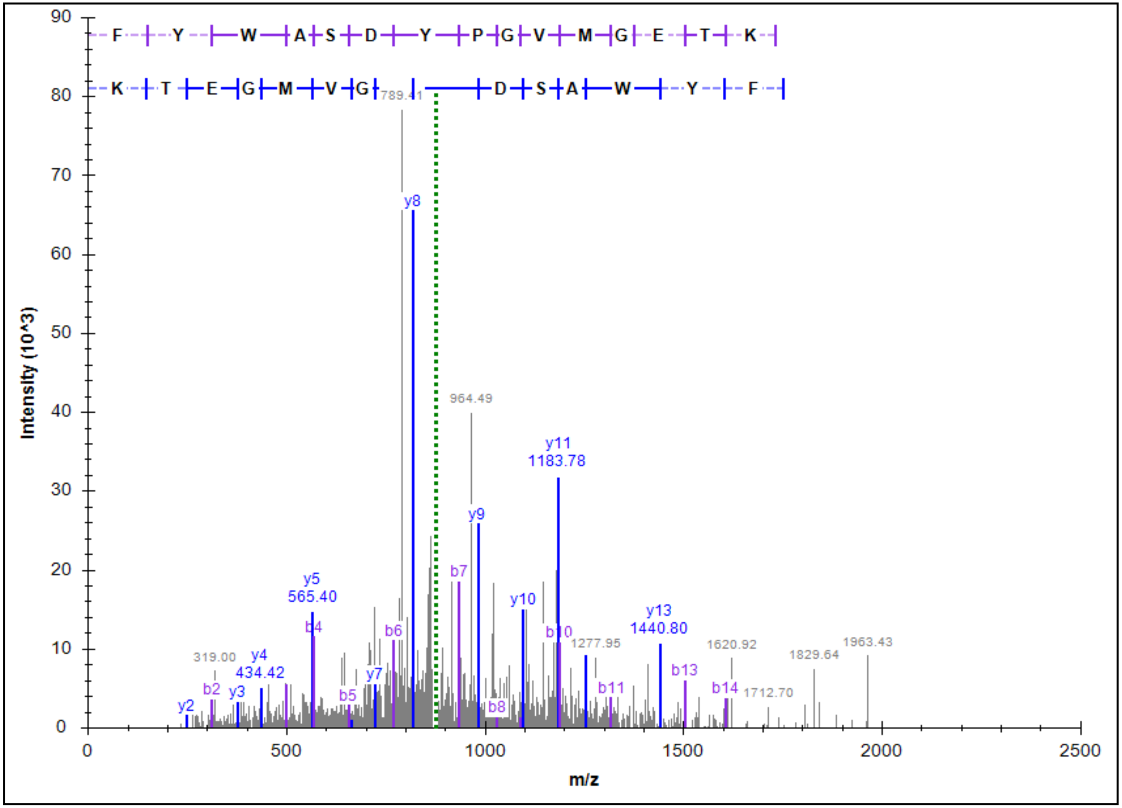
F Y W A S D Y P G V M G E T K
K T E G M V G D S A W Y F
789.41
964.49
y8
y9
y11
1183.78
y5
565.40
b7
y10
b4
b6
b10
1277.95
y13
1440.80
1620.92
1829.64
1963.43
319.00
y4
434.42
y7
b2
y3
y2
b5
b8
b11
b13
b14
1712.70
Intensity (10^3)
m/z
0
500
1000
1500
2000
2500
10
20
30
40
50
60
70
80
90
